# α-Synuclein Decreases the Abundance of Proteasome Subunits and Alters Ubiquitin Conjugates in Yeast

**DOI:** 10.3390/cells10092229

**Published:** 2021-08-28

**Authors:** Blagovesta Popova, Dajana Galka, Nicola Häffner, Dan Wang, Kerstin Schmitt, Oliver Valerius, Michael Knop, Gerhard H. Braus

**Affiliations:** 1 Department of Molecular Microbiology and Genetics, Institute for Microbiology and Genetics, University of Göttingen, 37077 Göttingen, Germany; dajana.galka@uni-goettingen.de (D.G.); n.haeffner@web.de (N.H.); dan.wang@uni-goettingen.de (D.W.); kschmit1@gwdg.de (K.S.); ovaleri@gwdg.de (O.V.); 2 Zentrum für Molekulare Biologie der Universität Heidelberg (ZMBH), DKFZ-ZMBH Alliance, Heidelberg University, 69120 Heidelberg, Germany; m.knop@zmbh.uni-heidelberg.de

**Keywords:** alpha-synuclein, Parkinson disease, protein aggregation, yeast, ubiquitin–proteasome system, protein homeostasis, posttranslational modifications, protein degradation

## Abstract

Parkinson’s disease (PD) is the most prevalent movement disorder characterized with loss of dopaminergic neurons in the brain. One of the pathological hallmarks of the disease is accumulation of aggregated α-synuclein (αSyn) in cytoplasmic Lewy body inclusions that indicates significant dysfunction of protein homeostasis in PD. Accumulation is accompanied with highly elevated S129 phosphorylation, suggesting that this posttranslational modification is linked to pathogenicity and altered αSyn inclusion dynamics. To address the role of S129 phosphorylation on protein dynamics further we investigated the wild type and S129A variants using yeast and a tandem fluorescent timer protein reporter approach to monitor protein turnover and stability. Overexpression of both variants leads to inhibited yeast growth. Soluble S129A is more stable and additional Y133F substitution permits αSyn degradation in a phosphorylation-independent manner. Quantitative cellular proteomics revealed significant αSyn-dependent disturbances of the cellular protein homeostasis, which are increased upon S129 phosphorylation. Disturbances are characterized by decreased abundance of the ubiquitin-dependent protein degradation machinery. Biotin proximity labelling revealed that αSyn interacts with the Rpt2 base subunit. Proteasome subunit depletion by reducing the expression of the corresponding genes enhances αSyn toxicity. Our studies demonstrate that turnover of αSyn and depletion of the proteasome pool correlate in a complex relationship between altered proteasome composition and increased αSyn toxicity.

## 1. Introduction

Parkinson’s disease (PD) is the second most common neurodegenerative disorder, affecting about 1% of the population older than 60 years. The cause of PD remains unknown although several risk factors, such as environmental influences, aging and genetic susceptibility, were identified to contribute to the onset of the pathogenic process [1]. PD is a genetically heterogeneous disorder with both familial and sporadic forms. Neuronal loss in the *substantia nigra*, which causes striatal dopamine deficiency, and intracellular inclusions termed Lewy bodies are neuropathological hallmarks of PD [2]. A major constituent of Lewy bodies is the protein alpha-synuclein (αSyn) [3]. Human αSyn is a pre-synaptic protein, containing 140 amino acids, and is abundantly expressed in the brain. αSyn is involved in modulation of synaptic activity, regulation of neurotransmitter release and regulation of cell differentiation [4]. αSyn was also reported to be localized in the nucleus, where it may promote neurotoxicity [5,6]. Missense mutations in αSyn alleles have been identified in rare familial inherited forms of PD [7,8,9,10,11]. Duplication and triplication of αSyn genes leading to overexpression of the gene product represent alternative genetic causes for PD [12,13]. These results suggest that an increase in the αSyn protein expression level could be sufficient to cause neurodegenerative disease.

Under pathological conditions αSyn accumulates to form oligomeric protofibrils that can further mature into different types of aggregate structures. This process is associated with the existence of a variety of intermediate species [14,15]. Aggregation of αSyn is assumed to constitute the central pathological process in synucleinopathies. Accumulating evidence suggests oligomeric or protofibrillar forms of αSyn, rather than mature aggregates and fibrils, to be responsible for neurotoxicity [16,17,18]. The pathological αSyn species are suggested to disrupt the molecular mechanisms of specific cellular processes, resulting in mitochondrial dysfunction, impairment of protein degradation, vesicle trafficking defects, disruption of vesicle-membrane fusion and inhibition of histone acetylation [1,19]. The molecular mechanisms how the aggregation process is initiated and how this protein causes pathogenic effects in a eukaryotic cell remains unknown. 

The accumulation of misfolded and aggregated αSyn indicates significant dysfunction in proteostasis in PD. The proteostasis network is overloaded by increasing amounts of toxic or aggregated αSyn species. The altered proteostasis in PD is dependent on the αSyn protein levels and on the impact of αSyn species on other components of the proteostasis network. The level of αSyn in the neuronal cells depends on the balance between the rates of αSyn synthesis, oligomerization, aggregation and clearance. A dysfunctional imbalance between these mechanisms can promote the formation and accumulation of toxic oligomeric and fibrillar species. The mechanism of αSyn clearance plays a major role in balancing the level of the protein and is a central question in understanding PD. Inefficient protein clearance as a result of impaired degradation pathways is sufficient to trigger neurotoxicity [20]. Soluble αSyn is degraded mainly through the ubiquitin-proteasome system (UPS), whereas autophagy represents the major pathway for the degradation of oligomeric species or aggregates of αSyn [21,22,23]. Various posttranslational modifications (PTMs) of αSyn, such as phosphorylation, ubiquitination, sumoylation or nitration, are primarily involved in modulating αSyn degradation by various proteolytic pathways [24,25,26,27]. These PTMs act as molecular switches that determine the preference of αSyn for a certain proteolytic process, indicating their important role in balancing the protein level of αSyn. 

Several studies support that proteasome dysfunction may contribute to the pathology of PD [28,29,30,31,32,33]. Reduced levels of proteasome subunits have been observed in PD patients. Several genes encoding proteasome subunits were downregulated in the *substantia nigra* of PD patients and linked to reduced levels of 20S core particles (CP) and 19S regulatory particles (RP) [34,35,36]. Overexpression of αSyn in cellular models of PD revealed decreased proteasomal function and accumulation of ubiquitin [37]. It was suggested that proteasome impairment is due to an altered proteasome composition rather than inhibition of individual peptidases within the proteasome complex [38] and that αSyn oligomeric species inhibit the UPS [39,40,41]. These studies support the hypothesis that αSyn interferes with the function of UPS, impairing its own clearance and also the degradation of other substrates. This leads to imbalances in cellular proteostasis. However, the specific role of the different proteasome subunits in PD pathology has not been thoroughly explored. 

The yeast *Saccharomyces cerevisiae* is an established reference cell used to understand the molecular basis of αSyn-induced toxicity, as well as other amyloidogenic proteins [42,43]. The availability of powerful genetic tools and resources, as well as the conservation of cellular pathways and functions with humans has established yeast as a model to improve our understanding of the molecular processes linked to neurodegenerative diseases, including PD. Numerous available yeast knock-out, overexpression or conditional libraries represent established powerful resources for large-scale genetic screening experiments and enabled the identification of multiple genes and pathways that affect αSyn-induced toxicity, which were further validated in more complex model organisms [44,45,46,47].

We used the yeast *Saccharomyces cerevisiae* as an eukaryotic reference cell and investigated the impact of αSyn expression and the phosphorylation-deficient variant S129A on overall protein homeostasis. Expression of the αSyn-encoding human *SNCA* gene, which has no homologue within the yeast genome, recapitulates several relevant aspects of PD in this cellular model. A systematic characterization of the interplay between αSyn and twelve subunits of the yeast proteasome revealed differential impacts of proteasome subunit depletion on αSyn toxicity. 

## 2. Materials and Methods

### 2.1. Yeast Transformation and Growth Conditions

The yeast strains and plasmids used in this study are listed in Table 1 and Table 2, respectively. Transformations of *S. cerevisiae* strains were performed by the standard lithium acetate protocol [48]. Yeast strains were grown at 30 °C in non-selective YEPD (yeast extract-peptone-dextrose). For integrative transformations, yeast strains were transformed with linearized integrative plasmid, allowing tandem integration into the *trp1* locus via homologous recombination. The number of integrated copies was verified by Southern blotting as previously described [23]. For all other experiments, cells were grown in synthetic complete dropout (SC) medium [49] lacking the relevant amino acids for selection, supplemented with 2% glucose, 2% raffinose or 2% galactose. Expression of *GAL1*-αSyn was induced by shifting overnight cultures from 2% raffinose to 2% galactose-containing SC selection medium. For downregulation of the *Tet*-promoter, the medium was supplemented with 10 µg/mL doxycycline. *GAL1* promoter shut-off experiments were performed after overnight induction of αSyn protein expression in 2% galactose medium in the presence or absence of 10 μg/mL doxycycline. Cells were pelleted, washed two times with water and shifted into SC medium supplemented with 2% glucose that represses the *GAL1* promoter. Cycloheximide chase experiments were performed by addition of 50 µg/mL cycloheximide to the selective SC medium. Cells were incubated further at 30 °C and samples were taken at the indicated time points. 

### 2.2. Spotting Assays

Yeast cells were pre-grown in selective SC medium containing 2% raffinose lacking the corresponding marker. After normalizing the cells to equal densities (*A_600_* = 0.1), 10-fold dilution series were prepared and 10 µL were spotted on SC-selection agar plates supplemented with either 2% glucose or 2% galactose. Where indicated, the plates were supplemented with 10 µg/mL doxycycline and incubated at 30 °C. 

### 2.3. Western Blot Analysis

Protein crude extract of all samples were prepared by breaking the cells mechanically with glass beads (∅ 0.25–0.5 mm, Carl Roth GmbH, Karlsruhe, Germany) in a buffer containing 1 mM EDTA pH 7.5, 50 mM DTT, 50 mM Tris-HCl pH 7.5 and 20 µL/mL protease inhibitor cocktail (Complete, EDTA-free, Roche Diagnostics GmbH, Germany) at 4 °C, and centrifuged at 13,000 rpm for 15 min to remove the glass beads and large cell debris. Protein concentration was determined using the Bradford protein assay and the protein samples were denatured in an SDS-sample buffer (50 mM Tris-HCl pH 6.8, 3% (*v*/*v*) β-mercaptoethanol, 2% (*w*/*v*) SDS, 1% (*v*/*v*) glycerol and 0.006% (*w*/*v*) bromophenol blue). For electrophoretic separations of the protein, equal amounts of protein extracts were subjected to 12% SDS-polyacrylamide-gel and transferred onto a nitrocellulose membrane. Blots were blocked in 5% skin milk powder in TBST buffer for 2 h and incubated with primary antibodies diluted in TBST buffer with 5% milk powder overnight. α/β/γSyn rabbit antibody (1:2000, Santa Cruz Biotechnology, Dallas, TX, USA), mouse anti phosphor Ser-129 αSyn antibody (1:2500, Wako Chemicals, Richmond, VA, USA), mouse anti-ubiquitin antibody (1:2000, Merch Millipore, Kenilworth, NJ, USA) and GAPDH mouse antibody (1:5000, Thermo Fisher Scientific, Waltham, MA, USA) were used. After three 10 min washes with TBST buffer, blots were incubated with secondary anti-mouse or anti-rabbit antibodies conjugated to peroxidase for 2 h at RT. After three 10 min washes with TBST, chemiluminescent reaction was performed with a detection substrate (44 µL 90 mM paracoumaric acid, 100 µL 2.5 M luminol, 6.2 µL H_2_O_2_, 2 mL 1 M Tris pH 8.5 and 18 mL H_2_O). Pixel density values for Western blot quantifications were obtained from TIFF files generated from digitized X-ray films (Kodak, Rochester, NJ, USA) and analyzed with the ImageJ software (NIH, Bethesda, MD, USA). Sample density values were normalized to the corresponding loading control. For quantification of the signals, at least three independent experiments were performed.

### 2.4. Fluorescence Microscopy and Quantifications

Yeast cells harboring αSyn-expressing plasmids were pre-grown in selective SC medium containing 2% raffinose at 30 °C overnight and transferred into galactose-containing SC medium +/− 10 µg/mL doxycycline for induction of αSyn expression overnight. Fluorescence images were obtained with 100× magnification using a Zeiss Observer. Z1 microscope (Zeiss, Oberkochen, Germany) equipped with a CSU-X1 A1 confocal scanner unit (YOKOGAWA), QuantEM:512SC digital camera (Photometrics, Tucson, AZ, USA) and SlideBook 6.0 software package (Intelligent Imaging Innovations, Göttingen, Germany). For *GAL1* promoter shut-off experiments, cells were pelleted, washed two times with water and shifted to the SC medium supplemented with 2% glucose to shut-off the *GAL1* promoter. The cells were visualized by fluorescence microscopy at time points 0 h, 2 h and 8 h. For quantification of the number of cells with inclusions, at least 200 cells were counted per strain and experiment. The number of cells displaying αSyn inclusions was referred to the total number of counted cells. 

Time-lapse fluorescence microscopy was performed with a CellASIC ONIX2 microfluidic devise (Merck, Kenilworth, NJ, USA) in 2Y04C-02 microfluidic yeast plates (Merck, Kenilworth, NJ, USA). The plate has four culture chambers for three-dimensional trapping of yeast cells that allows simultaneous monitoring of several strains. Cells were diluted to an OD_600_ of 0.1 and loaded into the microfluidic viewing chamber applying a pressure of 55.1 kPa for 5 s. Perfusion of fresh medium was conducted at 27.6 kPa. Images were acquired every hour at preset XY-positions using autofocusing with the Differential Interference Contrast (DIC) channel.

### 2.5. Flow Cytometry

Yeast cells were pre-grown in selective SC medium containing 2% raffinose at 30 °C to the mid-logarithmic phase. Expression of αSyn-tFT variants was induced for 6 h in SC medium supplemented with 2% galactose. Before flow cytometry measurements, the cells were washed and re-suspended in 50 mM trisodium citrate buffer, pH 7.0. Flow cytometry analysis was performed on a BD FACSCANTO II (Becton Dickinson, Franklin Lakes, NJ, USA). In total, 10,000 events were counted for each experiment. Data analysis was performed using the BD FACSDIVA software (Becton Dickinson, Franklin Lakes, NJ, USA).

### 2.6. Quantification and Statistical Analysis

Data were analyzed using GraphPad Prism 5 software (San Diego, CA, USA) and were presented as the mean ± SEM of at least three independent experiments. A *p*-value < 0.05 was considered to indicate a significant difference.

### 2.7. Sample Preparation for LC-MS Proteome Analysis

Yeast SILAC strain RH3493 was transformed with 2μ plasmids, harboring the corresponding αSyn genes without a tag, or an empty vector as a control. Overnight cultures were grown in 10 mL SC-Ura + 2% raffinose at 30 °C. Cells were harvested and transferred to a new 10 mL preculture in SC-Ura-Lys-Arg + 2% raffinose medium. Light, medium or heavy isotopically labeled lysin and arginine were added to the cultures at a concentration of 30 mg/L and 20 mg/L, respectively. The following stable isotopically labeled amino acids were used: ^13^C_6_-l-arginine HCl, ^13^C_6_^15^N_4_-l-arginine HCl, 4,4,5,5-D_4_-l-lysine HCl and ^13^C_6_,^15^N_2_-l-lysine HCl. The precultures were grown for 4 h at 30 °C, harvested by an OD_600_ of 0.8 and transferred into a 200 mL main culture (SC-Ura-Lys-Arg + 1% raffinose + 2% galactose), supplemented with the same combinations of light, medium or heavy isotopically labeled amino acids. The cultures were incubated overnight at 30 °C on a rotating shaker. Equal number of cells from each culture (OD = 2) were harvested and pooled together to get three pools (biological replicates) of differently labeled cultures, each one being a mix of αSyn+S129A+EV. Cell extracts were prepared as described in the Western blot analysis. A total of 60 µg protein from each protein pool was separated by 12% SDS-PAGE. Each gel lane was divided into 5 pieces, and proteins were subjected to in-gel digestion with trypsin according to the method of Shevchenko et al. [53]. After digestion and peptide elution the samples were resolved in 20 µL 2.8 % acetonitrile containing 0.1% formic acid. The tryptic peptides were then analyzed by LC-MS. 

### 2.8. LC-MS Analysis 

LC-MS analysis was performed as described previously using peptide solutions from trypsin-digested proteins [50]. MS/MS data were analyzed with the MaxQuant 1.5.1.0 software with the program’s default parameters, using a *Saccharomyces* *cerevisiae* protein database (UniProt, UP000002311, accessed date 3 April 2017). The digestion mode was trypsin/P, and a maximum of three missed cleavage sites was considered. Carbamidomethylation at cysteine was set as a fixed modification, and acetylation at the *N*-terminus, oxidation at methionine, and phosphorylation at serine, threonine and tyrosine were considered as variable modifications. Arg6 and Lys4 were defined as medium peptide labels and Arg10 and Lys8 as heavy peptide labels. Match between runs, Fourier transform-based mass spectrometer (FTMS) re-quantification and FTMS recalibration were enabled. For protein quantification, the minimum ratio count was 2. False discovery rates were calculated by MaxQuant and the filter was set to 0.01. MaxQuant output data were further processed using Perseus software [54].

### 2.9. BioID-SILAC Analysis

BioID-SILAC analysis was performed on the basis of Opitz et al. (2017). A scheme of the experimental procedure is presented in Figure S2. Cells were cultured overnight in selective medium containing 2% raffinose. A second preculture was inoculated from the first one and grown in selective medium for 6 h in the presence of stable isotope-labeled amino acids. Afterwards, the cells were diluted to OD_600_ = 0.1 and cultivated overnight in 200 mL selective medium containing 2% raffinose, 2% galactose, isotope-labeled amino acids and 10 µM biotin. Aliquots from each culture were taken for analysis by Western blot. Cells were harvested by centrifugation and equal amounts of cells expressing BirA*, αSyn-BirA* or S129A-BirA* (a total of 20 OD from each culture) were combined in a 1:1:1 ratio. Cells were resuspended in buffer containing 10 mM HEPES, 10 mM KCl, 1.5 mM MgCl_2_, 0.5 mM PMSF, 0.5 mM DTT and 20 µL/mL protease inhibitor cocktail (Complete, EDTA-free, Roche Diagnostics GmbH, Basel, Switzerland) and lysed mechanically by the use of glass beads. A total of 60 µg of the crude protein extract was used for proteome-based input control and directly separated by SDS-PAGE. The remaining extract was provided with SDS to a final concentration of 4%, vortexed, and then incubated for 5 min at 65 °C. The protein extract was cleared by centrifugation and the supernatant used for biotin affinity capture with StrepTactin Sepharose (gravity flow columns with 1 mL bed volume, #2–1202-001, IBA GmbH, Göttingen, Germany). The biotinylated proteins were eluted with a 10 mM biotin-containing buffer, precipitated by using a chloroform-methanol extraction protocol [55], resolved in 8M urea/2M thiourea and separated by SDS-PAGE. Whole lanes were subjected to in-gel digestion with trypsin for subsequent LC-MS analysis. SILAC quantification was performed with MaxQuant software and the output data were further processed using Perseus software.

## 3. Results

### 3.1. Tandem Fluorescent Protein Timer Monitoring Reveals That a Y133F Substitution Compensates the Deficiency in S129 Phosphorylation, which Normally Promotes Soluble αSyn Turnover

**Figure 1:**
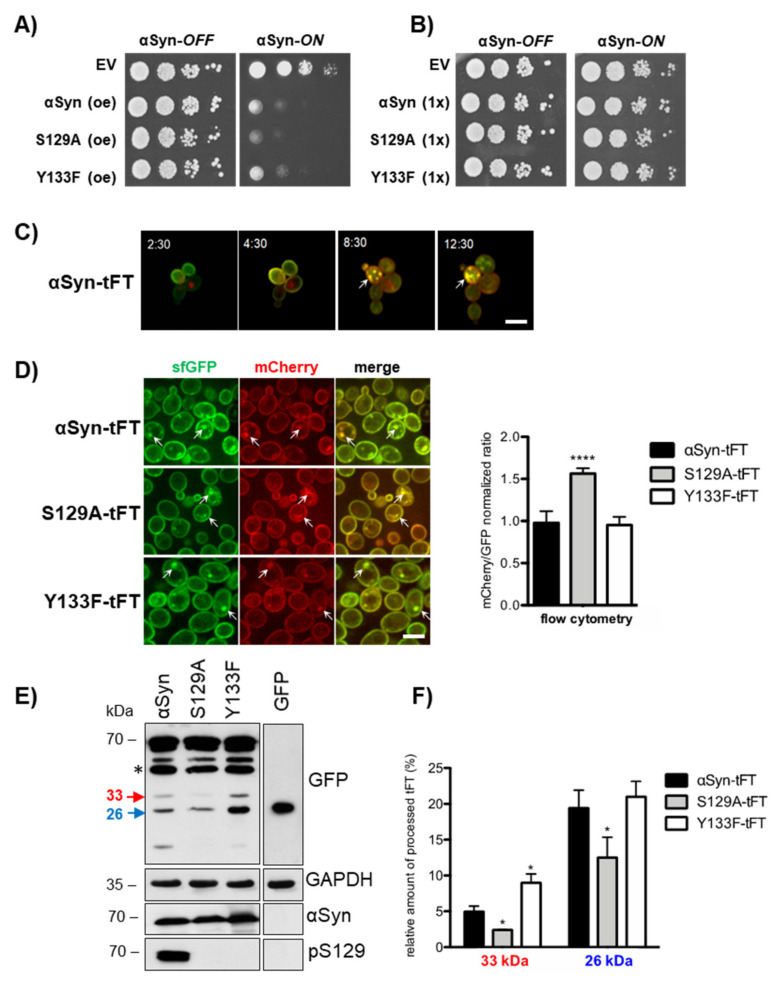
Phosphorylation at S129 promotes αSyn turnover. (**A**) Spotting test of yeast cells without (αSyn-OFF) or with (αSyn-ON) induction of *GAL1*-driven αSyn-GFP, S129A-GFP or Y133-GFP expression from 2 µ plasmids (oe—overexpression). Empty vector (EV) was used as a control. (**B**) Spotting test of corresponding yeast cells, expressing only a single integrated gene copy (1×) of *GAL1*-driven αSyn-tandem fluorescent timer (tFT), S129A-tFT, Y133-tFT or EV. (**C**) Fluorescence microscopy time series of yeast cells expressing single copy αSyn-tFT. The merge images show cells three-dimensionally trapped in a microfluidic device at the indicated time points after *GAL1* promoter-mediated induction. Intracellular inclusions are marked with arrows. Scale bar = 5 µm. (**D**) Fluorescence microscopy of strains expressing single-copy, tFT-tagged αSyn variants after 6 h induction of the *GAL1* promoter (left panel). Intracellular inclusions are marked with arrows. Scale bar = 5 µm. Fluorescence measurements with flow cytometry of the indicated strains (right panel). In total, 10,000 events were counted for each experiment. The significance of the differences was calculated with a *t*-test relative to αSyn-tFT (****, *p* < 0.0001, *n* = 6). (**E**) Western blot analysis of protein extracts from cells expressing single-copy, tFT-tagged αSyn variants or GFP as the control using GFP-antibody. The membrane was stripped and re-probed consecutively with αSyn antibody, S129 phosphorylation-specific αSyn antibody (pS129) or GAPDH antibody as the loading control. Only αSyn but no variant is phosphorylated at S129. The 33 kDa (red) fragment indicates proteasomal and the 26 kDa (blue) autophagy/vacuole-mediated degradation products. Asterisks indicate an mCherry∆N product resulting from mCherry hydrolysis during cell extract preparation [57]. (**F**) Densitometric analysis of the immunodetection of the specific tFT-αSyn degradation products. The relative intensity of the characteristic low molecular immunoblot bands was determined to examine the fate of the αSyn fusions. Band intensity relations within the same lane were quantified with ImageJ from anti-GFP immunoblot images. The relative amount of tFT degradation fragments to the total amount of loaded protein within one lane was calculated. The significance of the differences was determined with a *t*-test relative to αSyn (*, *p* < 0.05, *n* = 3).

The proteotoxicity of αSyn is dependent on its turnover, which is influenced by various posttranslational modifications. Overexpression of αSyn, as well as the S129A or Y133F variants that are deficient in phosphorylation or nitration, significantly inhibits yeast growth (Figure 1A). αSyn is abundantly phosphorylated at serine 129 and can be phosphorylated or nitrated at tyrosine 133. Phosphorylation at S129 promotes αSyn turnover by the 26S proteasome as well as the autophagy/vacuole pathways [26,56]. The *C*-terminal Y133 plays a major role in αSyn aggregate clearance. Y133 modification is required for the protective S129 phosphorylation as support for autophagy clearance, whereas non-modified Y133 promotes proteasome clearance [27]. 

Tandem fluorescent protein timer (tFT) fusions were employed as a tool to monitor the protein stability and turnover of the αSyn variants in vivo. tFT is a tandem fusion of two fluorescent proteins—mCherry and superfolder green fluorescent protein (sfGFP) with different kinetics of fluorophore maturation [58]. An sfGFP signal represents a young protein because it folds rapidly and becomes fluorescent shortly after protein synthesis. An mCherry signal corresponds to a more aged protein, because it requires a longer time to fold and become fluorescent. The ratio of mCherry/sfGFP fluorescence intensities represents the average age of the corresponding protein and decreases as the degradation rate of the mCherry–sfGFP fusions increases. Thus, the degradation of αSyn protein, its localization and the age of the protein pool can be followed by quantification of the red and green signals as measures for protein age. The tFT was fused to the C-terminus of αSyn or the variants deficient in phosphorylation or nitration (S129A or Y133F). Yeast strains were generated with genomically integrated single copies of the αSyn-tFT gene variants in order to avoid variations in the plasmid copy number between cells. Expression of αSyn from one gene copy is below the toxicity threshold and does not inhibit yeast growth (Figure 1B). Fluorescence microscopy was used to visualize the age-dependent subcellular localization of αSyn-tFT (Figure 1C). Cells were trapped in a microfluidic device and the maturation of the fluorescent timer was followed within single cells with time. The pool of mCherry-sfGFP molecules was mostly green-fluorescent shortly after protein induction and gradually acquired red fluorescence over time, demonstrating that the ratio of red to green fluorescence is a function of the age of the protein pool. We measured the mCherry/sfGFP fluorescence ratios of tFT-tagged proteins 6 h after *GAL1* induction using flow cytometry (Figure 1D). A similar ratio for Y133F-tFT in comparison to wildtype αSyn supports equal cellular stability. In contrast, the S129A-tFT protein exhibited a significantly higher mCherry/sfGFP ratio than the (wildtype, indicating increased stability. 

Processed tFT fragments can be exploited as a marker of proteasomal degradation [59]. The degradation pattern of αSyn-tFT fusion proteins was analyzed by immunoblot analysis, where only αSyn but neither the S129A nor the Y133F variant were phosphorylated at serine-129 (Figure 1E). A fraction of sfGFP from the tFT resists degradation due to the stability of the GFP fold, which results in accumulation of tFT fragments in the cell. The existence of a 33 kDa band is attributed to incomplete proteasomal degradation, whereas a 26 kDa band is characteristic for vacuolar degradation of the protein [59,60]. The band intensities can be directly correlated with the degradation pathway responsible for protein turnover. Increased accumulation of the 33 kDa band as indicator of proteasomal degradation was observed for Y133F-tFT in comparison to the wildtype, which agrees with previous findings, suggesting that non-modified Y133 promotes aggregate clearance by the proteasome [27]. Quantification of the relative 33 kDa and 26 kDa intensities revealed a significant reduction in both the 33 kDa as well as 26 kDa band upon expression of S129A-tFT fusion in comparison to the wildtype, indicative of reduced overall turnover of the protein by the proteasome and vacuole, respectively (Figure 1F). This novel approach for a quantitative assessment of the αSyn dynamics in living cells corroborates that the potential to phosphorylate S129 is a major determinant for αSyn homeostasis that can be compensated by a Y133F substitution, which allows αSyn degradation in a phosphorylation-independent manner.

### 3.2. αSyn Expression Changes the Yeast Proteome and Reduces the Proteasome Subunit Levels

Protein homeostasis in eukaryotic cells depends on two highly conserved degradative pathways, the ubiquitin-26S-proteasome system and autophagy mediated by double-membraned autophagosome vesicles, which are targeted to the vacuole/lysosome compartments. A coordinated and complementary crosstalk between these systems becomes critical under proteostatic stress [61]. A genome-wide screen with yeast strain collections comprising conditional alleles of essential genes revealed multiple modulators of αSyn toxicity [47]. The most prominent categories were connected with protein homeostasis. Ubiquitin-dependent protein degradation was the second largest category of genes that affect αSyn-induced toxicity. 

**Figure 2:**
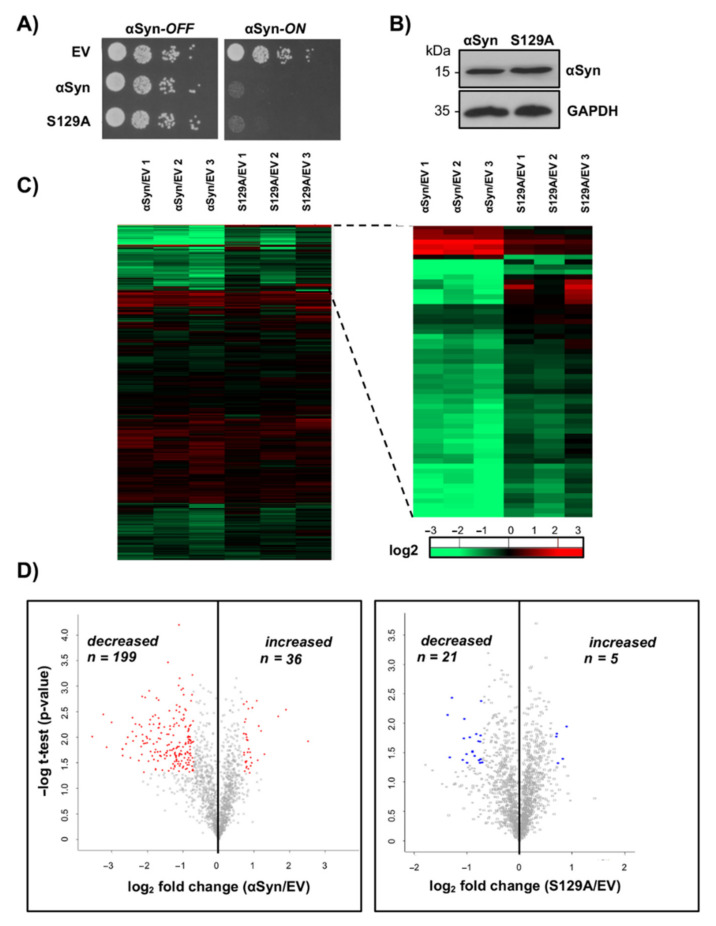
Expression of αSyn changes significantly the relative protein abundance, whereas S129A has less impact on the yeast proteome. (**A**) Growth assay of yeast cells expressing *GAL1*-driven αSyn or S129A from a 2 µ plasmid with an empty vector (EV) as a control. Cells were spotted in 10-fold dilutions on selective plates containing glucose (αSyn-*OFF*) or galactose (αSyn-*ON*). (**B**) Western blot analysis with protein crude extracts of cells expressing αSyn or S129A after overnight induction in SILAC strain RH3493. GAPDH was used as a loading control. (**C**) Heatmap of the protein enrichment relative to the empty vector control (EV) (*n* = 3; isotope label-swap replication). Colors indicate the levels of enrichment: green—downregulation; red—upregulation; black—non-significantly regulated. The right panel represents magnification of the indicated section. (**D**) Volcano plot analysis. Proteins were ranked according to their statistical *p*-value (y-axis) and their relative abundance ratio (log2-fold change) (x-axis). The threshold for significance was *p* ≤ 0.05 and log2-fold change ≤ −0.7 or ≥ 0.7. Proteins with significantly changed abundance upon αSyn or S129A expression are colored in red or blue, respectively.

We used a proteomics approach that enables quantitative monitoring of derailed protein abundances in response to expression of αSyn, which is normally phosphorylated at serine-129 compared to the S129A variant, which cannot be phosphorylated (Figure 1E). We sought to unravel disturbances in the protein degradation pathways due to αSyn expression, including the contribution of S129 phosphorylation to this effect. Stable isotope labeling of amino acids in cell culture (SILAC) was used for quantitative proteome comparisons analyzed with LC-MS. The yeast SILAC strain was transformed with the 2 µ plasmid, harboring αSyn encoding gene without a tag, S129A, or an empty vector as the negative control. Expression of both the αSyn and S129A variant inhibited yeast growth considerably (Figure 2A). αSyn expression was induced in a galactose-containing medium overnight. Immunoblot analysis revealed similar expression levels for αSyn and S129A (Figure 2B).

Proteins of three independent cell lines (expressing either αSyn, its S129A variant, or the empty vector control) were labeled in culture with the light, medium or heavy isotope variants of lysine and arginine. Afterwards, an equal number of cells were combined for a quantitative proteome analysis by LC-MS/MS. Label swap was performed in order to exclude expression artefacts due to incomplete incorporation of isotopic amino acids. Three independent biological replicate pools of αSyn, S129A and the control cells were prepared and processed for LC-MS/MS analysis. The differential protein abundances between the samples were calculated by comparing the intensity differences of the triplets of isotope-labeled peaks in MS. Stringent thresholds were applied to decide on the inclusion of proteins for analysis. Comparisons were made across the three independent experimental replicates to establish reproducibility. Only proteins identified in all nine samples were considered for analysis. The threshold for significance was set to 60% enriched (log_2_ SILAC ratio = 0.7 and *p* < 0.05), determined by Rab guanosine triphosphatase Ypt1, a known suppressor of αSyn toxicity that is conserved from yeast cells to dopaminergic neurons [44,62]. 

In total, 1559 proteins were quantified (Table S1). The abundance of 235 proteins significantly differed in αSyn-expressing cells compared to the control cell line (Figure 2C,D, Table S2). Among them, 199 proteins had decreased abundance and 36 proteins increased abundance upon αSyn expression. Expression of the more stable S129A had a less significant impact on the yeast proteome. The abundance of 26 proteins significantly differed in comparison to the control cell line (Figure 2C,D, Table S3). Functional enrichment analysis of the proteins with significantly changed abundance was performed on the basis of gene ontology (GO) terms and Munich Information Center for Protein Sequences (MIPS) categories. The most significantly affected biological pathway upon αSyn expression in comparison to the control involving proteins with decreased abundance was protein degradation (*p* = 3.00 × 10^−8^) (Table S4). Processes attributed to αSyn toxicity, such as electron transport (*p* = 1.29 × 10^−7^), oxidative stress (*p* = 5.04 × 10^−7^), energy generation (*p* = 2.51 × 10^−5^) or protein folding (*p* = 4.00 × 10^−4^), were significantly enriched in the functional analysis. 

Expression of the more stable S129A revealed significant functional enrichment of mitochondrial proteins (Table S5). Analysis of the proteins with increased levels revealed functional enrichment of the proteins associated with fatty acid metabolism, endocytosis, or ER to Golgi transport. Functional enrichment for cellular localization was similar for αSyn and S129A and included ER, Golgi, vacuole and mitochondria, four subcellular localizations known to be affected by αSyn [44,63,64,65]. Known modulators of αSyn toxicity were identified, such as Ypt1 [44], Yhb1 [27], Acc1 [66], COX5A [67] and SOD1 [68]. This confirms that the proteomics analysis captured meaningful biological events associated with αSyn toxicity, and reveals a strong link between αSyn toxicity and a changed cellular proteome. 

Ten proteins functioning as proteasome subunits are among the protein degradation category, which is most significantly affected upon αSyn expression. Six proteins represent components of the catalytic 20S core particle (Pre3, Pre5, Pre7, Pre8, Pre9 and Pre10). Four proteins are components of the 19S regulatory particle and include the ubiquitin receptor Rpn10, the lid subunits Rpn8 and Rpn12 and the base subunit Rpn13 of the 26S proteasome. Expression of S129A, which cannot be phosphorylated, resulted in a lower impact on the abundance of these proteasome subunits (Table 3). This result demonstrates that αSyn expression decreases the abundance of multiple proteasome subunits. αSyn toxicity is therefore connected with changes in the proteasome as a key player in eukaryotic protein homeostasis. Phosphorylation at S129 significantly enhanced the general impact on the yeast proteome and reduced the abundance of proteasome subunits.

### 3.3. Downregulation of Genes for Proteasome Subunits Enhances αSyn Toxicity 

A systematic comparison of the interplay of differential expression of proteasome genes and αSyn was conducted. We examined the effects of downregulation of different proteasome subunits on αSyn toxicity or aggregation. The yeast 26S proteasome has 33 distinct subunits encoded by 28 essential genes and 5 non-essential genes [69]. It consists of the proteolytically active barrel-like 2.5 MDa 20S core particle (CP). The CP is composed of two outer heptameric α-rings (α1–α7) and two inner β-rings (β1–β7). The 20S CP provides unspecific ATP-independent protease activity. The CP is capped with one or two 19S regulatory particles (RP). The 19S RP is assembled by a lid containing up to ten non-ATPase subunits and a base consisting of six AAA^+^ ATPase subunits (Rpt1–Rpt6) and two non-ATPase subunits. The lid provides specificity and coordinates substrate recognition and removal of the polyubiquitin chains of the labeled substrates. The ATPase base subunits form a hetero-hexameric structure that mediates substrate unfolding, CP gate opening and translocation of substrates into the catalytic barrel of the CP [70]. 

Twelve genes encoding subunits of the 26S proteasome were selected among the conditional alleles of essential genes in the *Tet*-Promoters Hughes collection (yTHC) [71]. The proteasome subunits Pre5, Rpn5 and Rpn11 were previously identified as modulators of αSyn toxicity [47], and Pre3, Pre5 and Rpn8 were significantly downregulated in the proteomic analysis. We expanded the analysis with additional components of the proteasome, since αSyn may trigger molecular events not captured in the original assays. Therefore, three genes encoding subunits of the lid of the 19S RP (Rpn5, Rpn8 and Rpn11), three encoding subunits of the base of the 19S RP (Rpt2, Rpt4 and Rpt6) and six genes encoding proteasome CP subunits (Pre1, Pre3, Pre4, Pre5, Pre6 and Pre8) were further analyzed.

**Figure 3:**
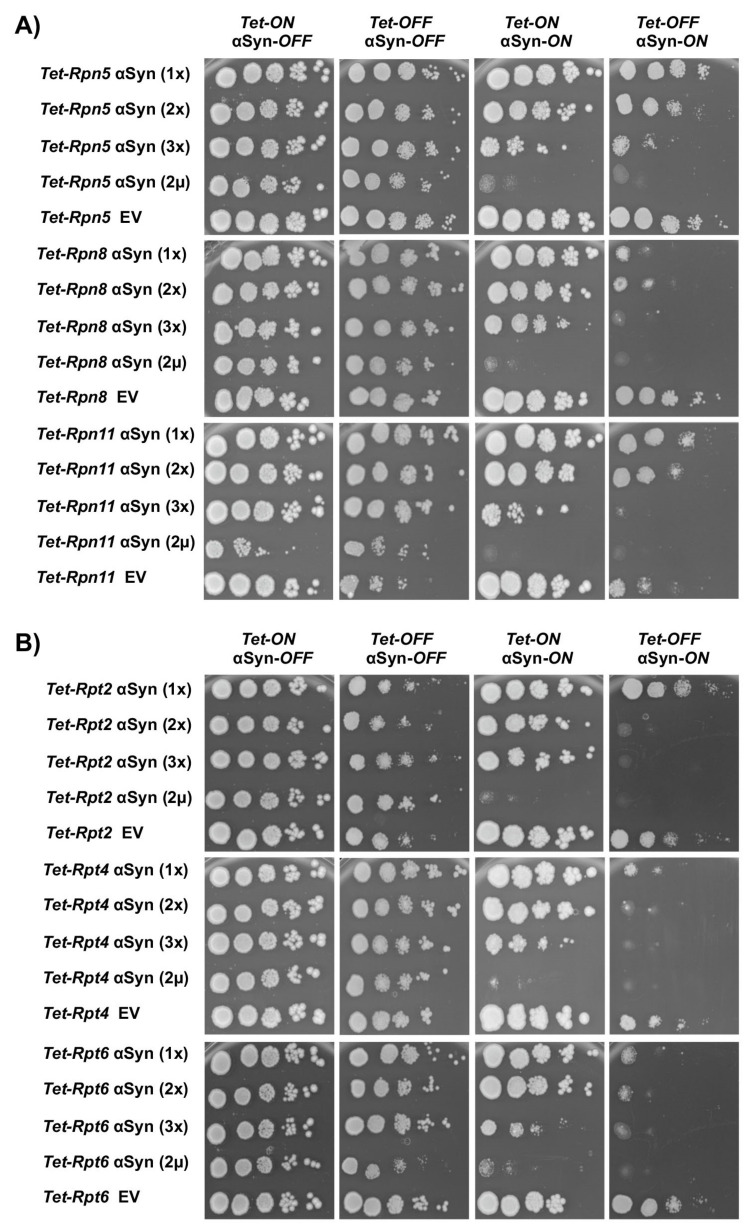
Growth effect on yeast cells upon interaction between αSyn and the *Tet* alleles of the essential genes encoding the lid (**A**) or base (**B**) subunits. Growth assays of yeast cells expressing *GAL1*-driven αSyn-GFP from one (1×, two (2×) or three (3×) gene copies or overexpressed from a 2 µ plasmid with an empty vector (EV) as a control. Cells were spotted in 10-fold dilutions on selective plates containing glucose (αSyn-*OFF*) or galactose (αSyn-*ON*), in the presence (*Tet-OFF*) or absence (*Tet-ON*) of 10 μg/mL doxycycline that represses the *Tet* promoter. The plates were incubated at 30 °C for 5 days.

**Figure 4:**
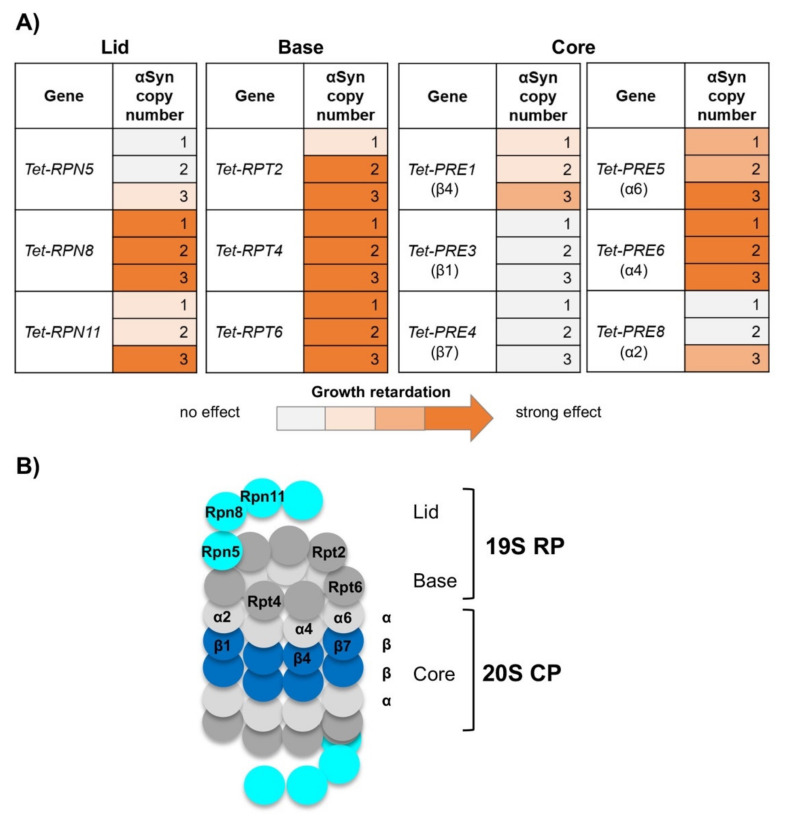
Downregulation of the gene expression of multiple proteasome subunits significantly enhances αSyn toxicity. (**A**) Heatmap representing the genetic interactions upon downregulation of the proteasome genes and αSyn expression. A strong synthetic sick phenotype was observed upon downregulation of multiple proteasome subunits genes. The strongest response was detected upon downregulation of the lid and base subunits. The growth inhibition increased with increasing dose of αSyn. (**B**) Schematic structure of the yeast 26S proteasome, consisting of the 19S regulatory particle (RP) and 20S core particle (CP). The proteasome subunits from (**A**) are indicated within the structure.

αSyn was expressed at different levels at and below the toxicity threshold from a regulatable *GAL1* promoter, in order to screen for synthetic interactions. Yeast strains were constructed with single, double or triple integrations of the αSyn-GFP encoding gene at the single *trp1* locus of the yTHC strains (Table 1). In these strains, the endogenous promoter of each essential gene is replaced with a *Tet*-titratable promoter in the genome. The promoter can switch off the gene expression by addition of doxycycline to the yeast growth medium, resulting in protein depletion. Growth assays were performed by downregulation of the expression level of the proteasome genes and by normal expression levels. Downregulation of the genes encoding the proteasome lid subunits Rpn5, Rpn8 and Rpn11 resulted in synthetic sick phenotypes in presence of αSyn (Figure 3A). The growth retardation correlated with gene dosage. Expression from one or two copies of αSyn revealed a weak growth defect, whereas expression from three copies or plasmid-borne from the 2 µ plasmid was synthetic sick for *RPN5* and synthetic lethal for *RPN8* and *RPN11* upon downregulation of the *Tet* promoter. Growth assays performed upon downregulation of the base subunit genes *Tet-RPT2*, *Tet-RPT4* and *Tet-RPT6* showed even stronger genetic interactions (Figure 3B). Expression of αSyn from two gene copies was already enough to cause a synthetic lethal phenotype. Expression of αSyn in conditional strains of proteasome core subunits showed the strongest synthetic sick effect upon downregulation of *Tet-PRE5* and *Tet-PRE6* (Figure S1). In summary, the downregulation of different proteasome genes had a broad range of effects on αSyn toxicity (Figure 4). The strongest impact was observed upon downregulation of genes encoding regulatory particle subunits, where downregulation of five out of six genes significantly increased the αSyn toxicity, even at a low copy number.

### 3.4. αSyn Interacts with the Base Subunit Rpt2 

**Figure 5:**
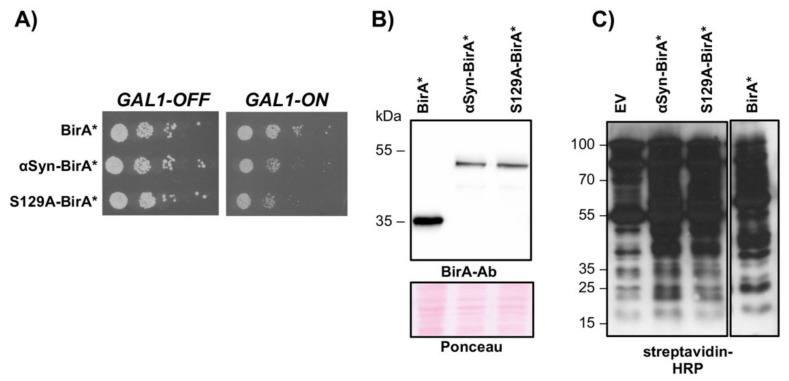
BirA* biotinylates endogenous protein in yeast cells. (**A**) Growth assay of yeast cells, expressing *GAL1*-driven BirA*, αSyn-BirA* and S129A-BirA* from a high copy plasmid. (**B**) Immunodetection of proteins from (**A**) using a BirA-specific antibody. Ponceau staining of the lanes is shown as a loading control. (**C**) Protein biotinylation, detected with horseradish peroxidase (HRP)-coupled streptavidin, is elevated in cells expressing BirA*-fusion proteins or BirA* alone in comparison to the empty vector (EV). Protein expression was induced in galactose-containing medium in the presence of biotin.

Biotin IDentification (BioID) proteomics was employed for analysis of the protein microenvironment of αSyn in yeast to explore whether there is any proximity between αSyn and subunits of the 26S proteasomes within the cell. BioID is a unique unbiased method identifying the physiologically relevant protein proximities or interactions in living cells [72]. This technique uses a biotin ligase fused to a bait protein to label the proximal proteins in vivo. The *E. coli*-derived promiscuous biotin ligase BirA* was genetically fused to αSyn or its variant S129A and the respective fusion genes were expressed in yeast cells. BirA* covalently labels the neighboring proteins with biotin at the exposed lysine residues ~10 nm apart. Cells expressing free BirA* were used as the negative control. Expression of the fusion proteins inhibited yeast growth (Figure 5A), but to a lesser extent in comparison to expression of the non-tagged protein (Figure 2A). Immunoblot detection with a BirA antibody revealed similar expression levels for αSyn-BirA* and S129A-BirA* (Figure 5B). Analysis with HRP-conjugated streptavidin showed enhanced protein biotinylation upon expression of BirA*-fusion proteins or BirA* in the presence of biotin (Figure 5C). Enrichment quantification of biotinylated BioID candidates was done applying SILAC labeling that enabled relative quantification of the proteins from different cultures in one batch. Three different cell cultures were separately cultivated and supplemented with light, medium or heavy stable isotope variants of lysine and arginine in the presence of biotin. Following the SILAC strategy, a similar number of cells from the respective cultures were pooled directly after cultivation and further processed as one batch according to the described BioID workflow (Figure S2). The relative enrichment of proteins from the BirA*-fusion-expressing strains in comparison to the control was evaluated using SILAC ratios. BioID-captured proteins were considered significantly enriched when they were at least 60% enriched (log_2_ SILAC ratio = 0.7; one-sample *t*-test < 0.05, *n* = 3) compared with the BirA* control. 

In total, 44 proteins were identified that are significantly enriched upon αSyn-BirA* or S129A-BirA* expression in comparison to the BirA* control (Table S6). Most of the BioID interactions in the cellular proximity of αSyn were associated with the plasma membrane and to cellular compartments or transport vesicles (Table S6 marked in yellow). This is in accordance to the finding that overexpression of αSyn in yeast interferes with intracellular trafficking and results in abnormal vesicle accumulation, clustering and toxicity [62]. In addition, αSyn is close to a number of signaling components (Table S6 marked in blue). This includes the factors involved in αSyn proteotoxicity as Sec4, which is required for vesicular docking at the plasma membrane and is an ortholog of human Rab8a that interacts with αSyn in the rodent brain [73]. The small GTPase Ras2 is also involved in αSyn toxicity [74]. There are also single αSyn BioID interactions to DNA or RNA binding, transcription or cell cycle proteins (Table S6 marked in green).

There were two BioID hits for αSyn to proteins with potential protein stability functions. This includes a putative Mindy deubiquitinase with unknown cellular function and one subunit of the 26S proteasome. The Rpt2 subunit of the RP is part of the base and was identified as a novel proteasome subunit that is proximate to an αSyn subpopulation. This suggests that αSyn might directly or indirectly physically interact with the 19S RP and might interfere with assembly or disassembly of 26S proteasomes. In contrast to αSyn, the abundance of Rpt2 in the biotinylated fraction eluted upon expression of S129A was lower and below the threshold for significance (log_2_ SILAC ratio = 0.49), which suggests that the base interaction includes primarily the phosphorylated form of αSyn.

### 3.5. Downregulation of Genes for Proteasome Subunits Results in Different Outcomes of αSyn Inclusion Formation

**Figure 6:**
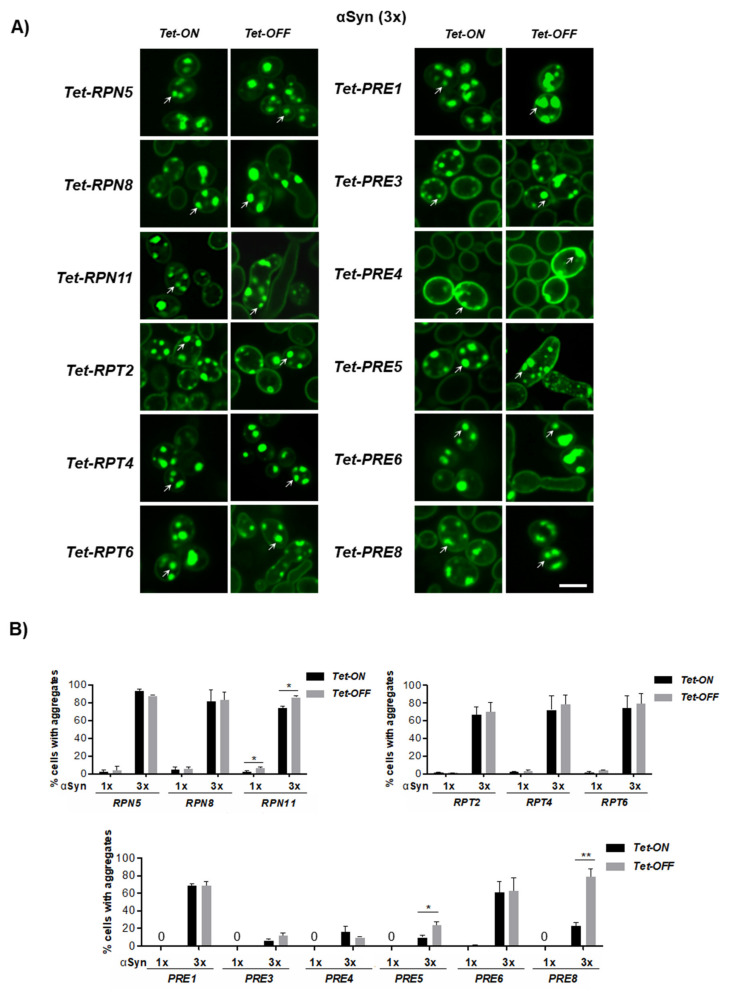
Inclusion formation of αSyn upon downregulation of the proteasome gene expression. (**A**) Fluorescence microscopy of yeast cells expressing αSyn-GFP from three gene copies in strains with *Tet* alleles of proteasome genes after 16 h induction in a galactose-containing medium. *Tet* promoter was downregulated by addition of 10 µg/mL doxycycline to the growth medium simultaneously with the induction of αSyn expression. Intracellular inclusions are marked with arrows. Scale bar = 5 μm. (**B**) Quantification of the percentage of cells displaying αSyn-GFP inclusions. “0” indicates cells without inclusion. The significance of the differences was determined with a *t*-test (*, *p* < 0.05; **, *p* < 0.01; *n* = 3).

Our data suggest that soluble phosphorylated αSyn is not only degraded by the 26S proteasome but also interacts with the base of the RP and might disturb the function of the 26S proteasome or even result in disassembly of the core and regulatory particles. Therefore, the impact of αSyn expression combined with downregulation of the genes for subunits of the base or the lid of the 19S RP or of the 20S CP was further assessed. *Tet* strains expressing αSyn-GFP from one copy and three gene copies were used. αSyn-GFP expression was induced overnight in the presence or absence of doxycycline and fluorescence microscopy was used to determine the number of cells with inclusions. Cells expressing αSyn-GFP from one gene copy showed no aggregation or a low number of cells with inclusions, whereas expression from three gene copies resulted in an increased number of cells with inclusions (Figure 6A). Downregulation of the *Tet* promoter could result in elongated cells but did not affect αSyn-GFP inclusion formation in most of the strains (Figure 6B). Significant increase in inclusion formation was observed only upon downregulation of *Tet-RPN11*, *Tet-PRE5* and *Tet-PRE8*. These results indicate a differential impact of proteasome subunit depletion on αSyn-GFP inclusion formation. 

**Figure 7:**
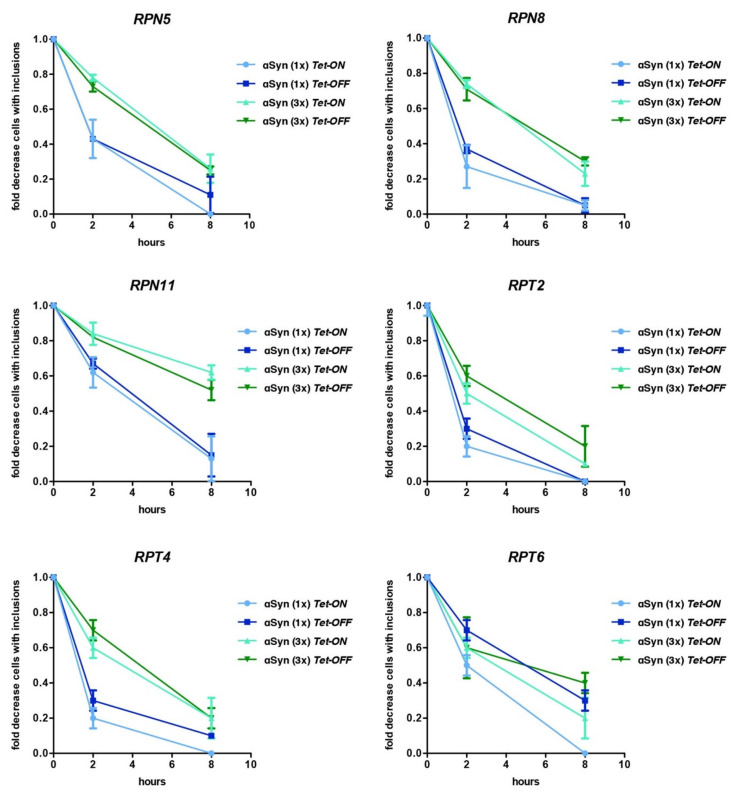
Downregulation of the proteasome gene expression does not significantly affect αSyn aggregate clearance. αSyn aggregate clearance after *GAL1*-promoter shut-off of cells expressing αSyn-GFP from one or three gene copies. Cells were grown overnight in galactose-containing medium for induction of protein expression and then shifted to a glucose medium that represses the *GAL1* promoter. The cells with inclusions were counted at time points 0 h, 2 h and 8 h after *GAL1*-promoter shut-off and normalized to time point zero. The *Tet* promoter was downregulated by addition of 10 µg/mL doxycycline to the growth medium. Values represent the mean ± SEM of three independent experiments.

The kinetics of αSyn aggregate clearance was investigated in *Tet* strains of genes encoding RP subunits to assess, whether downregulation of different proteasome subunits changes the ability of yeast cells to clear inclusion. Promoter shut-off studies were performed where αSyn expression was induced overnight in galactose-containing medium in presence and absence of doxycycline, followed by promoter shut-off in glucose-containing medium that represses the *GAL1* promoter (Figure 7). The removal of aggregates was monitored with fluorescence microscopy. The clearance of αSyn inclusions was not significantly affected by the expression levels of the tested proteasome genes up to 8 h after promoter shut-off, since inclusions were cleared similarly upon *Tet-ON* or *Tet-OFF*. However, there were differences in the αSyn degradation kinetics among strains. Aggregates were cleared most efficiently in the *Tet-RPT2* and *Tet-RPT4* strains. The kinetics of clearance was similar for the *Tet-RPN5, Tet-RPN8* and *Tet-RPT6* strains. Clearance of αSyn inclusions in *Tet-RPN11* was much more inefficient than in the other *Tet* strains. The differences in aggregate clearance between strains are probably due to the exchange of the endogenous promoter of the proteasome genes with the *Tet* promoter that misregulates the native gene expression.

These results indicate that the strong enhancement of αSyn toxicity upon downregulation of multiple proteasome genes is not accompanied by increased αSyn inclusion formation and represents a distinct outcome. The analysis revealed differences in the αSyn degradation kinetics among strains. Pronounced effects were observed upon downregulation of *RPN11*, representing an important modulator of αSyn turnover.

### 3.6. αSyn Diminishes the Pool of Ubiquitin Conjugates upon Downregulation of Tet-RPN11

**Figure 8:**
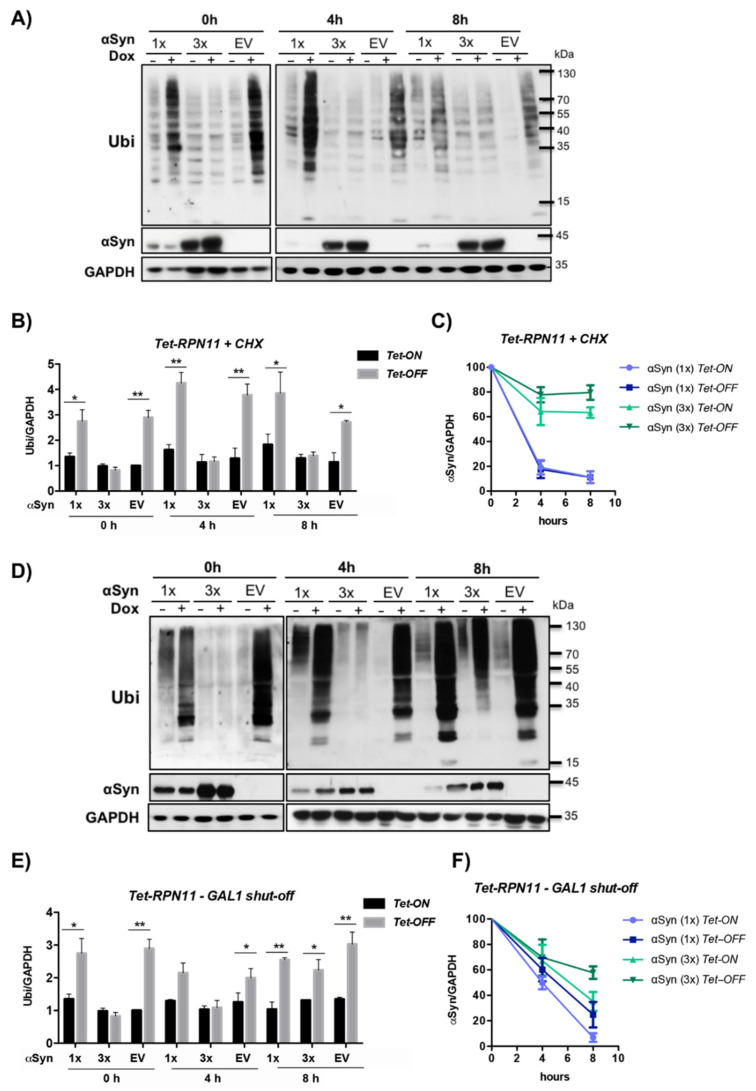
High level of αSyn decreases the pool of ubiquitinated substrates upon downregulation of *Tet-RPN11*. (**A**) Western blot analysis of *Tet-RPN11* mutant strains treated with cycloheximide. Strains harboring one (1×) or three (3×) gene copies of αSyn or an empty vector (EV) as a control were grown overnight in a galactose medium to induce αSyn expression. The *Tet* promoter was downregulated by addition of 10 µg/mL doxycycline to the growth medium. The next day, cells were treated with 50 µg/mL cycloheximide to stop de novo protein synthesis. Immunoblotting analysis was performed at the indicated time points after addition of cycloheximide with ubiquitin (Ubi) or αSyn antibodies. GAPDH was used as a loading control. (**B**) Densitometric analysis of the immunodetection of the ubiquitin conjugates relative to the GAPDH loading control. The ubiquitin/GAPDH ratio was normalized to the ratio of EV (*Tet*-ON) at 0 h. The significance of the differences was calculated with a *t*-test (*, *p* < 0.05; **, *p* < 0.01, *n* = 3). (**C**) Densitometric analysis of the immunodetection of αSyn relative to the GAPDH loading control. The αSyn/GAPDH band ratio was normalized to the corresponding ratio at 0 h and presented as percentage. Values represent the mean ± SEM of three independent experiments. (**D**) Recovery of the depleted ubiquitin pool is accompanied by αSyn degradation. Western blot of *Tet*-*RPN11* mutant strains after *GAL1* promoter shut-off. Cells were grown overnight in a galactose medium to induce αSyn expression and transferred to a glucose medium that repress the *GAL1* promoter. Immunoblot analysis was performed at the indicated time points after the shift to a glucose medium with ubiquitin, αSyn and GAPDH antibody as a loading control. (**E**) Densitometric analysis of the immunodetection of ubiquitin conjugates relative to the GAPDH loading control. The ubiquitin/GAPDH ratio was normalized to the corresponding ratio of EV-Dox at 0 h. The significance of the differences was calculated with a *t*-test (*, *p* < 0.05; **, *p* < 0.01, *n* = 3). (**F**) Densitometric analysis of the immunodetection of αSyn relative to the GAPDH loading control. The αSyn/GAPDH band ratio was normalized to the corresponding ratio at 0 h and presented as a percentage. Values represent the mean ± SEM of three independent experiments.

The de-ubiquitinating enzyme (DUB) Rpn11 is an important protein for ubiquitin recycling, because it removes ubiquitin from a substrate, which is tagged for degradation [75,76]. Therefore, we assessed the effect of αSyn expression on the steady-state level of ubiquitinated proteins (Figure 8A). Changes in the ubiquitin pool by downregulation of *Tet-RPN11* and different levels of αSyn expression were examined. Yeast cells were treated with the translational inhibitor cycloheximide to arrest de novo protein synthesis after overnight αSyn expression in the presence and absence of doxycycline. This procedure permits visualization of the degradation kinetics of the steady-state population of cellular proteins. Samples were taken at 0 h, 4 h and 8 h after cycloheximide treatment. The levels of high molecular weight ubiquitin conjugates were evaluated by immunoblot analysis and compared between strains, expressing αSyn from one gene copy, three gene copies or a vector as a control in the presence or absence of doxycycline. 

The ubiquitin pool showed severe differences when one copy or three copies of αSyn integrated into *Tet-RPN11* were compared. The pool of ubiquitinated substrates was significantly increased upon doxycycline treatment in the empty vector control, as well as in *Tet-RPN11* harboring one copy of αSyn (Figure 8B). This result corroborates previous findings revealing accumulation of ubiquitinated proteins in non-functional proteasomes [77,78,79]. Especially mutations within genes for deubiquitinating enzymes are essential for ubiquitin homeostasis [75,76,80]. An increase in the αSyn protein levels resulted in a significant decrease of the ubiquitin conjugates upon downregulation of *Tet-RPN11* in comparison to the empty vector control or low levels of αSyn under the same conditions. Cycloheximide chase experiments resulted in slight accumulation of ubiquitinated proteins upon *Tet-OFF* after a 4 h chase. After an 8 h chase, the pool of ubiquitinated proteins was decreased due to limited availability of free ubiquitin in the cells upon inhibition of translation [79]. The level of αSyn was assayed over time (Figure 8C). The degradation of the protein was inhibited upon high levels of αSyn expression, suggesting that *RPN11* promotes the turnover of soluble monomeric αSyn. 

We addressed whether the changes in the ubiquitin pool upon high-level expression of αSyn can be rescued after depletion of the protein. αSyn expression was induced overnight in the presence or absence of doxycycline, and the cells were transferred to glucose-containing medium to shut-off the *GAL1* promoter. Significant changes in the ubiquitin pool with decreasing levels of αSyn over time could be observed (Figure 8D,F). With decreasing αSyn levels, the accumulation of ubiquitinated proteins upon downregulation of *Tet-RPN11* after 8 h promoter shut-off increased and was similar to that of the empty vector control. These results reveal a correlation between the changes in the pool of ubiquitinated proteins and the level of αSyn and show that the depletion of ubiquitinated proteins upon downregulation of *Tet-RPN11* is a reversible process.

### 3.7. αSyn Increases the Pool of Ubiquitin Conjugates upon Downregulation of Tet-RPT2

Analysis of the ubiquitin pool was performed upon downregulation of *Tet-RPT2* as base subunit with proximity to αSyn in the BioID experiments. Rpt2 was also chosen for further analysis because of the strong synthetic sick growth phenotype upon downregulation of the gene, as well as its function. The proteasomal base subunit Rpt2 functions as an ATPase. The released energy is used to unfold and translocate substrates through the open channel into the 20S proteasome [81]. Rpt2 is essential for the assembly of the regulatory 19S complex, since it associates with other ATPases and thereby promotes their specific placement in the complex [82]. Ubiquitin pool analysis in *Tet-RPT2* should clarify whether αSyn has the same impact or it interferes differently with specific proteasome subunits. 

**Figure 9:**
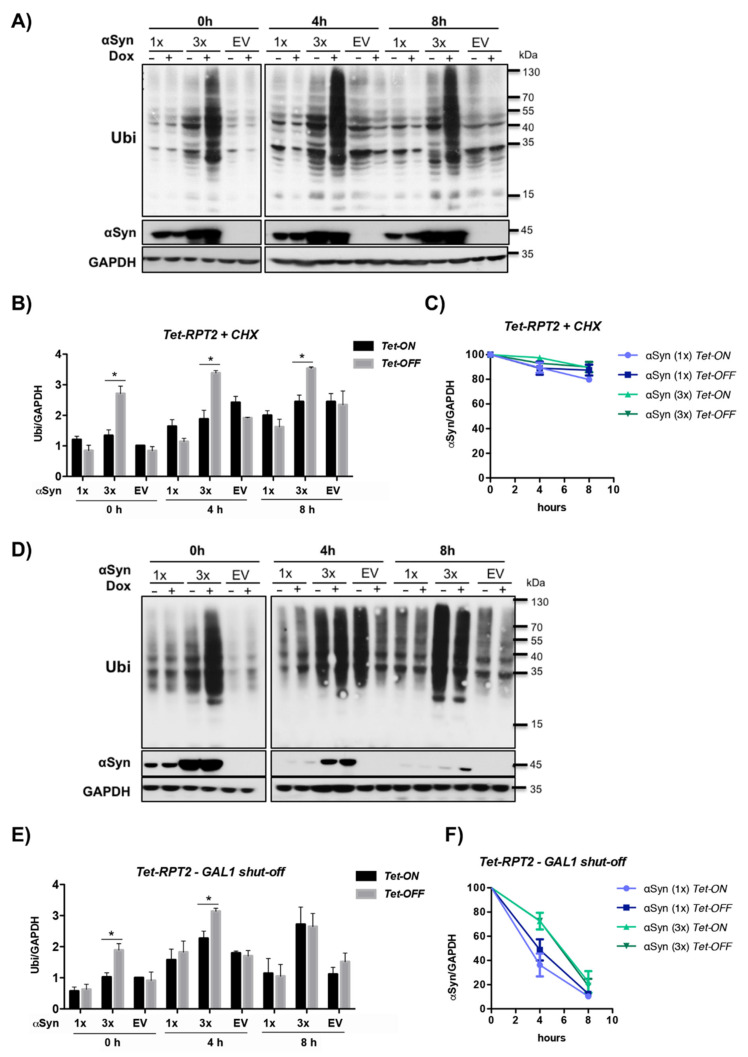
High level of αSyn increases the pool of ubiquitinated substrates upon downregulation of *Tet-RPT2*. (**A**) Western blot analysis of *Tet-RPT2* mutant strains treated with cycloheximide. Strains harboring one (1×) or three (3×) gene copies of αSyn or empty vector (EV) as a control were grown overnight in galactose medium to induce αSyn expression. The *Tet* promoter was downregulated by addition of 10 µg/mL doxycycline to the growth medium. Afterwards, cells were treated with 50 µg/mL cycloheximide to stop de novo protein synthesis. Immunoblotting analysis was performed at the indicated time points after addition of cycloheximide with ubiquitin (Ubi) or αSyn antibodies. GAPDH was used as a loading control. (**B**) Densitometric analysis of the immunodetection of high-molecular weight ubiquitin conjugates relative to the GAPDH loading control. The ubiquitin/GAPDH ratio was normalized to the ratio of EV (*Tet*-ON) at 0 h. The significance of the differences was calculated with a *t*-test (*, *p* < 0.05; *n* = 3). (**C**) Densitometric analysis of the immunodetection of αSyn relative to the GAPDH loading control. The αSyn/GAPDH band ratio was normalized to the corresponding ratio at 0 h and presented as a percentage. Values represent the mean ± SEM of three independent experiments. (**D**) Immunoblot analysis of the *Tet*-*RPT2* mutant strains after *GAL1* promoter shut-off. Cells were grown overnight in a galactose medium to induce αSyn expression and transferred to a glucose medium that repress the *GAL1* promoter. Immunoblot analysis was performed at the indicated time points after the shift to glucose medium with ubiquitin, αSyn or GAPDH antibody as a loading control. (**E**) Densitometric analysis of the immunodetection of ubiquitin conjugates relative to the GAPDH loading control. The ubiquitin/GAPDH ratio was normalized to the corresponding ratio of EV-Dox at 0 h. The significance of the differences was calculated with a *t*-test (*, *p* < 0.05; *n* = 3). (**F**) Densitometric analysis of the immunodetection of αSyn relative to the GAPDH loading control. The αSyn/GAPDH band ratio was normalized to the corresponding ratio at 0 h and presented as a percentage. Values represent the mean ± SEM of three independent experiments.

Yeast *Tet-RPT2* strains with one or three gene copies of αSyn or vector as a control were processed similarly as the *Tet-RPN11* strains. After overnight induction of αSyn expression in the presence or absence of doxycycline, cycloheximide chase was performed, and samples analyzed at 0 h, 4 h and 8 h after the treatment. The levels of ubiquitin conjugates were analyzed by immunoblotting (Figure 9A). The effect of downregulation of *Tet-RPT2* on the level of ubiquitinated proteins was opposite to that observed for *Tet-RPN11.* Downregulation of *Tet-RPT2* had no effect on the accumulation of ubiquitinated proteins in the absence of αSyn or at low expression levels (Figure 9B). In contrast to *Tet-RPN11*, the ubiquitin pool was drastically increased when *Tet*-*RPT2* was downregulated. Cycloheximide treatment resulted in a general increase in the levels of ubiquitinated proteins upon *Tet-ON*; however the impact of a high level of αSyn upon downregulation of the *Tet*-promoter was unchanged. Interestingly, cycloheximide completely blocked αSyn protein degradation in the *Tet-RPT2* strain (Figure 9C). No differences in αSyn turnover could be observed in cells, expressing one or three copies of αSyn grown in the presence or absence of doxycycline. 

The *GAL1* promoter shut-off experiments were carried out similar to the experiments with the *Tet-RPN11* strain to follow the changes of the ubiquitin pool upon αSyn depletion (Figure 9D,E). Protein expression was induced overnight in the presence or absence of doxycycline, and the cells were transferred to glucose-containing medium to shut-off the *GAL1* promoter. With decreasing αSyn levels at 8 h post promoter shut-off (Figure 9F), the difference in the ubiquitin pool between *Tet-ON* and *Tet-OFF* (3 × αSyn) disappeared; however, yeast cells were not able to process the accumulated ubiquitinated proteins and the levels were significantly higher compared to the empty vector control or cells expressing one copy of αSyn. 

These results reveal that high levels of αSyn lead to different cellular responses upon downregulation of the proteasome base subunits *RPT2* or the lid subunit *RPN11.* Whereas αSyn strongly inhibits the proteasome ability to degrade ubiquitin conjugates upon downregulation of *RPT2*, it has the opposite effect upon downregulation of *RPN11.* This suggests that αSyn disturbs the proteasome function via multiple pathways, resulting in alteration of ubiquitin homeostasis. 

## 4. Discussion

**Figure 10:**
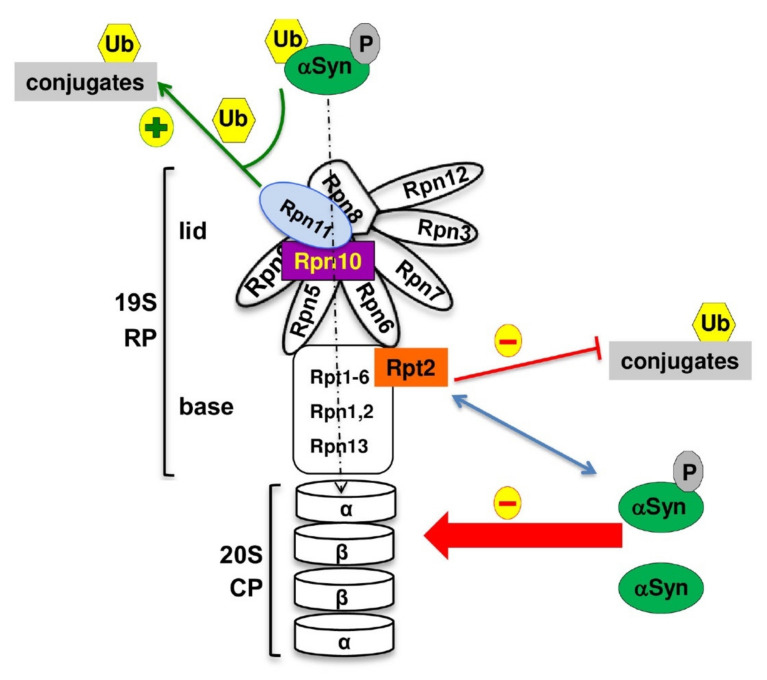
αSyn expression significantly reduces the proteasome pool. Model of the 26S proteasome consisting of a 19S regulatory particle (RP) and 20S core particle (CP). Ubiquitinated (Ub) substrates associate with the proteasome mediated by the RP subunits Rpn10 and Rpn13. The deubiquitinating enzyme Rpn11, which is part of the lid, removes polyubiquitin chains from the substrates to further promote their degradation. Phosphorylation at S129 promotes turnover of soluble αSyn by the proteasome. The base subunit Rpt2 promotes opening of the entry pore of the 20S CP, which enables protein substrates to be translocated into the catalytic channel for degradation. Regulation of CP–RP interactions is essential for substrate-specific proteasome degradation function. Phosphorylated αSyn is in proximity to the Rpt2 base subunit, which presumably causes proteasome dysfunction and an altered pool of ubiquitin conjugates. αSyn significantly reduces the abundance of the proteasome subunits, which could be due to 26S disassembly and subsequent degradation of the proteasome subcomplexes.

The main finding of this study is that αSyn cannot only be degraded in its soluble form by the 26S proteasome but is also present in the proximity of the 19S regulatory particle close to the Rpt2 base subunit or the Rpn10 ubiquitin receptor (Figure 10). Quantitative proteomic analysis revealed a significant disruption of protein homeostasis upon expression of αSyn. αSyn affected most significantly the protein degradation pathway and reduced the abundance of ten proteasome subunits. This effect correlates with αSyn turnover, since the more stable non-phosphorylatable S129A variant has a smaller impact on the yeast proteome. The interaction of αSyn to the base subunit Rpt2 involves primarily the phosphorylated form of αSyn. Proteasome stress caused by depletion of single proteasome subunits significantly enhanced αSyn toxicity, with the strongest impact observed for the downregulation of RP subunits. Our data support that αSyn can cause different types of proteasome stresses, which alter the proteasome abundance and the degradation of ubiquitinated proteins. Downregulation of the *RPN11* gene for the lid ubiquitin isopeptidase in combination with high levels of αSyn results in depletion of the pool of cellular ubiquitinated proteins. In contrast, downregulation of *RPT2* encoding one of the base ATPases in combination with high levels of αSyn increased the pool of cellular ubiquitinated proteins. It is yet elusive whether the interaction of an αSyn subpopulation with the proteasome base can interfere with the assembly, disassembly or the stability of 26S proteasomes.

Proteasome abundance is determined by the balance between the synthesis and degradation of the proteasome particles. Proteaphagy is a process of autophagic turnover of proteasomes to reduce the abundance as well as defective particles [83]. A coordinated and complementary crosstalk between proteasome degradation and synthesis can be critical under proteostatic stress [84,85,86,87]. Binding of αSyn to Rpt2 or Rpn10 might reduce the connection between the lid and the base and/or between the 19S regulatory with the 20S core particle. Accurate 26S proteasome assembly is essential to control the cell cycle, gene expression or the response to oxidative stress [88]. Binding of mutated αSyn [89,90] or aggregated forms of αSyn [39,91] with the proteasome and impairment of proteasome activity has been reported previously. Thus, proteasomal dysfunction by αSyn can induce selective removal of inactive or damaged proteasome particles by proteaphagy and reduce the abundance of proteasomes in the cell. The phosphorylation state of αSyn at S129 probably tunes the interaction and/or proteasome impairment and results in differences in proteasome abundance. 

Rpn11 is the metallo-isopeptidase activity of the lid of the 19S RP that removes ubiquitin from substrates to be degraded by the 26S proteasome. The DUB activity of this intrinsic proteasome subunit promotes degradation. The inactivation of the deubiquitinating activity of Rpn11 prevents the degradation of proteasome substrates [75,76]. Rpn11 is located in close proximity to the ubiquitin receptor Rpn10 and to the substrate entry pore formed by the ATPase ring within the proteasome RP close to the ATPase ring [92]. Active translocation of the substrate by the ATPases presumably presents the chain to Rpn11 that cuts polyubiquitin en bloc at the base of the chain. Therefore, downregulation of Rpn11 results in accumulation of ubiquitinated proteins. Upon high levels of αSyn, the pool of ubiquitinated proteins was depleted. αSyn might affect the translocation of the substrates into the catalytic core and promote degradation of ubiquitin along with the conjugated substrates, thus escaping the DUB activity of Rpn11 and causing depletion of the cellular ubiquitin pool and ubiquitin wasting. Depletion of ubiquitin causes toxicity in yeast [79]. Loss of function of Rpn11 in aging *Drosophila melanogaster* caused reduced 26S proteasome activity, a premature age-dependent accumulation of ubiquitinated proteins and enhanced neurodegenerative phenotype [93], whereas overexpression of Rpn11 restored the 26S proteasome activity, resulting in lifespan extension. This suggests Rpn11 as a key factor in neurodegeneration and implies that increasing the amount of the lid subunit Rpn11 may suppress αSyn toxicity.

αSyn was found in close proximity to Rpt2, which is one of the six nonredundant ATPase subunits of the base. It has a unique role during 26S proteasome formation and activation, opening the entry pore of the 20S CP and enabling protein substrates to be translocated into the proteolytic channel for degradation [81]. Downregulation of this subunit caused strong enhancement of toxicity even by low expression levels of αSyn. αSyn induced significant accumulation of ubiquitinated proteins when Rpt2 was depleted, suggesting proteasome dysfunction. Consistently, depletion of Rpt2 resulted in mice in accumulation of αSyn and development of Lewy Body-like inclusions [94], and strongly induced neurodegeneration and PD-like symptoms in *Drosophila* [95]. Significant alteration in the expression of proteasome subunits was reported in the spinal cord of the A30P αSyn variant in mice [96]. Depletion of RP subunits might change the cellular subunit stoichiometry and could interfere with 26S proteasome assembly, leading to increased numbers of defective proteasomes [97]. The impairment of the proteasome activity connected to neurodegeneration may be mediated by physical contact of αSyn to the regulatory particle, resulting in blockage within the proteasome complex at crucial sites and alterations in proteasome composition or stability.

The establishment of a tandem fluorescent protein timer technique allowed us to monitor the cytosolic turnover of αSyn in vivo. αSyn undergoes numerous post-translational modifications (PTMs), such as phosphorylation, ubiquitination, sumoylation, nitration or acetylation [98,99,100,101,102,103]. αSyn can be degraded by the UPS, which is located in nucleus and cytoplasm, or autophagosome-mediated by the vacuole/lysosome as an alternative pathway [104]. Both clearance pathways are participating in αSyn degradation in yeast using αSyn PTMs as molecular tag determinants for channeling the protein to different pathways. PTMs influence αSyn aggregation and toxicity and additionally modulate the degradation of the protein [25]. Previously we have shown that αSyn inclusions are cleared by a combination of autophagy and vacuolar protein degradation [23]. Posttranslational modifications of αSyn shift the ratio of clearance between autophagy and the UPS degradation pathways [26,27,56]. Phosphorylation at S129 is a major determinant for protein degradation. C-terminal tyrosine 133 (Y133) plays a major role in αSyn degradation by supporting the protective S129 phosphorylation for aggregate clearance by autophagy and by promoting proteasome clearance of soluble αSyn [27]. The tandem fluorescent protein timer technique revealed that soluble pS129 is preferentially degraded by the proteasome. Thus, αSyn phosphorylation is directly involved in maintaining αSyn protein homeostasis. Surprisingly, expression of S129A evoked less significant changes in the yeast proteome. The number of identified proteins with changed abundance was reduced in comparison to the changes induced by αSyn. Additionally, S129A triggered a less significant reduction in the levels of proteasome subunits. This observation indicates that the depletion of the proteasome pool correlates with αSyn turnover. Phosphorylated αSyn (pS129) is an important molecular switch that directs the protein to the proteasome. Increased αSyn turnover promoted by pS129 triggered downregulation of the levels of proteasome subunits. This indicates a complex crosstalk and negative feedback between αSyn posttranslational modifications and the ubiquitin–proteasome system. This study provides novel links between αSyn phosphorylation and protein turnover as well as αSyn proteasome interaction and inhibition as further insight into understanding the complex causes of Morbus Parkinson.

## Figures and Tables

**Table 1:** Yeast strains used in this study.

Name	Genotype	Source
W303-1A	*MATa; ura3-1; trp1D2; leu2-3_112; his3-11; ade2-1; can1-100*	EUROSCARF
RH3851	*W303-1A: ura3-1*::*GAL1-SNCA-mCherry-sfGFP-ADH1-kanMX* (one genomic copy)	This Study
RH3852	*W303-1A: ura3-1*::*GAL1-SNCA(S129A)-mCherry-sfGFP-ADH1-kanMX* (one genomic copy)	This Study
RH3853	*W303-1A: ura3-1*::*GAL1-SNCA(Y133F)-mCherry-sfGFP-ADH1-kanMX* (one genomic copy)	This Study
RH3493	*MATα, ura3-52, trp1::hisG, Δarg4::loxP, Δlys1::loxP*	[50]
R1158	*MATa URA3::CMV-tTA his3-1 leu2-0 met15-0 (kanMX4:G418R)*	yTHC collection, Horizon Discovery, UK
yTHC-916	*Tet*-*RPN5* in R1158	yTHC collection, Horizon Discovery, UK
yTHC-481	*Tet*-*RPN8* in R1158	yTHC collection, Horizon Discovery, UK
yTHC-719	*Tet*-*RPN11* in R1158	yTHC collection, Horizon Discovery, UK
yTHC-76	*Tet*-*RPT2* in R1158	yTHC collection, Horizon Discovery, UK
yTHC-24	*Tet*-*RPT4* in R1158	yTHC collection, Horizon Discovery, UK
yTHC-681	*Tet*-*RPT6* in R1158	yTHC collection, Horizon Discovery, UK
yTHC-761	*Tet*-*PRE1* in R1158	yTHC collection, Horizon Discovery, UK
yTHC-345	*Tet*-*PRE3* in R1158	yTHC collection, Horizon Discovery, UK
yTHC-1012	*Tet*-*PRE4* in R1158	yTHC collection, Horizon Discovery, UK
yTHC-564	*Tet*-*PRE5* in R1158	yTHC collection, Horizon Discovery, UK
yTHC-657	*Tet*-*PRE6* in R1158	yTHC collection, Horizon Discovery, UK
yTHC-545	*Tet*-*PRE8* in R1158	yTHC collection, Horizon Discovery, UK
RH3854	*Tet-RPN5* in *R1158*; 1 genomic copy of *GAL1-SNCA-GFP* in *trp1* locus	This Study
RH3855	*Tet-RPN5* in *R1158*; 2 genomic copies of *GAL1-SNCA-GFP* in *trp1* locus	This Study
RH3856	*Tet-RPN5* in *R1158*; 3 genomic copies of *GAL1-SNCA-GFP* in *trp1* locus	This Study
RH3857	*Tet-RPN5* in *R1158;* pME5037 (EV) in *trp1* locus	This Study
RH3858	*Tet-RPN8* in *R1158*; 1 genomic copy of *GAL1-SNCA-GFP* in *trp1* locus	This Study
RH3859	*Tet-RPN8* in *R1158*; 2 genomic copies of *GAL1-SNCA-GFP* in *trp1* locus	This Study
RH3860	*Tet-RPN8* in *R1158*; 3 genomic copies of *GAL1-SNCA-GFP* in *trp1* locus	This Study
RH3861	*Tet-RPN8* in *R1158;* pME5037 (EV) in *trp1* locus	This Study
RH3862	*Tet-RPN11* in *R1158*; 1 genomic copy of *GAL1-SNCA-GFP* in *trp1* locus	This Study
RH3863	*Tet-RPN11* in *R1158*; 2 genomic copies of *GAL1-SNCA-GFP* in *trp1* locus	This Study
RH3864	*Tet-RPN11* in *R1158; 3 genomic copies of GAL1-SNCA-GFP* in *trp1* locus	This Study
RH3865	*Tet-RPN11* in *R1158;* pME5037 (EV) in *trp1* locus	This Study
RH3866	*Tet-RPT2* in *R1158*; 1 genomic copy of *GAL1-SNCA-GFP* in *trp1* locus	This Study
RH3867	*Tet-RPT2* in *R1158*; 2 genomic copies of *GAL1-SNCA-GFP* in *trp1* locus	This Study
RH3868	*Tet-RPT2* in *R1158*; 3 genomic copies of *GAL1-SNCA-GFP* in *trp1* locus	This Study
RH3869	*Tet-RPT2* in *R1158*; pME5037 (EV) in *trp1* locus	This Study
RH3870	*Tet-RPT4* in *R1158*; 1 genomic copy of *GAL1-SNCA-GFP* in *trp1* locus	This Study
RH3871	*Tet-RPT4* in *R1158*; 2 genomic copies of *GAL1-SNCA-GFP* in *trp1* locus	This Study
RH3872	*Tet-RPT4* in *R1158*; 3 genomic copies of *GAL1-SNCA-GFP* in *trp1* locus	This Study
RH3873	*Tet-RPT4* in *R1158*; pME5037 (EV) in *trp1* locus	This Study
RH3874	*Tet-RPT6* in *R1158*; 1 genomic copy of *GAL1-SNCA-GFP* in *trp1* locus	This Study
RH3875	*Tet-RPT6* in *R1158*; 2 genomic copies of *GAL1-SNCA-GFP* in *trp1* locus	This Study
RH3876	*Tet-RPT6* in *R1158*; 3 genomic copies of *GAL1-SNCA-GFP* in *trp1* locus	This Study
RH3877	*Tet-RPT6* in *R1158*; pME5037 (EV) in *trp1* locus	This Study
RH3878	*Tet-PRE1* in *R1158*; 1 genomic copy of *GAL1-SNCA-GFP* in *trp1* locus	This Study
RH3879	*Tet-PRE1* in *R1158*; 2 genomic copies of *GAL1-SNCA-GFP* in *trp1* locus	This Study
RH3880	*Tet-PRE1* in *R1158*; 3 genomic copies of *GAL1-SNCA-GFP* in *trp1* locus	This Study
RH3881	*Tet-PRE1* in *R1158*; pME5037 (EV) in *trp1* locus	This Study
RH3882	*Tet-PRE3* in *R1158*; 1 genomic copy of *GAL1-SNCA-GFP* in *trp1* locus	This Study
RH3883	*Tet-PRE3* in *R1158*; 2 genomic copies of *GAL1-SNCA-GFP* in *trp1* locus	This Study
RH3884	*Tet-PRE3* in *R1158*; 3 genomic copies of *GAL1-SNCA-GFP* in *trp1* locus	This Study
RH3885	*Tet-PRE3* in *R1158*; pME5037 (EV) in *trp1* locus	This Study
RH3886	*Tet-PRE4* in *R1158*; 1 genomic copy of *GAL1-SNCA-GFP* in *trp1* locus	This Study
RH3887	*Tet-PRE4* in *R1158*; 2 genomic copies of *GAL1-SNCA-GFP* in *trp1* locus	This Study
RH3888	*Tet-PRE4* in *R1158*; 3 genomic copies of *GAL1-SNCA-GFP* in *trp1* locus	This Study
RH3889	*Tet-PRE4* in *R1158*; pME503 (EV) in *trp1* locus	This Study
RH3890	*Tet-PRE5* in *R1158*; 1 genomic copy of *GAL1-SNCA-GFP* in *trp1* locus	This Study
RH3891	*Tet-PRE5* in *R1158*; 2 genomic copies of *GAL1-SNCA-GFP* in *trp1* locus	This Study
RH3892	*Tet-PRE5* in *R1158*; 3 genomic copies of *GAL1-SNCA-GFP* in *trp1* locus	This Study
RH3893	*Tet-PRE5* in *R1158*; pME5037 (EV) in *trp1* locus	This Study
RH3894	*Tet-PRE6* in *R1158*; 1 genomic copy of *GAL1-SNCA-GFP* in *trp1* locus	This Study
RH3895	*Tet-PRE6* in *R1158*; 2 genomic copies of *GAL1-SNCA-GFP* in *trp1* locus	This Study
RH3896	*Tet-PRE6* in *R1158*; 3 genomic copies of *GAL1-SNCA-GFP* in *trp1* locus	This Study
RH3897	*Tet-PRE6* in *R1158*; pME5037 (EV) in *trp1* locus	This Study
RH3898	*Tet-PRE8* in *R1158*; 1 genomic copy of *GAL1-SNCA-GFP* in *trp1* locus	This Study
RH3899	*Tet-PRE8* in *R1158*; 2 genomic copies of *GAL1-SNCA-GFP* in *trp1* locus	This Study
RH3900	*Tet-PRE8* in *R1158*; 3 genomic copies of *GAL1-SNCA-GFP* in *trp1* locus	This Study
RH3901	*Tet-PRE8* in *R1158*; pME5037 (EV) in *trp1* locus	This Study

**Table 2:** Plasmids used in this study.

Name	Description	Source
p426	*2µm, URA3, GAL1, CYC1, AmpR*	[51]
pME5037	pRS305 *(LEU2, GAL1, CYC1, AmpR)* with *TRP1*	[47]
pME5038	pME5037 with *GAL1-SNCA-GFP*	[47]
pME5039	p425 *(2µm, LEU2, CYC1, AmpR)* with *GAL1-SNCA-GFP*	[47]
pME3760	p426*-GAL1-SNCA*	[23]
pME3759	p426*-GAL1-GFP*	[23]
pME5320	p426*-GAL1-SNCA(S129A)*	This study
pME5321	pFA6a-*GAL1-SNCA-mCherry-sfGFP-kanMX*	This study
pME5322	pFA6a-*GAL1-SNCA(S129A)-mCherry-sfGFP-kanMX*	This study
pME5322	pFA6a-*GAL1-SNCA(Y133F)-mCherry-sfGFP-kanMX*	This study
pME4480	pME2787-MET25-BirA*	[52]
pME5324	p426-GAL1-αSyn-BirA*	This study
pME5325	p426-GAL1-SNCA(S129A)-BirA*	This study

**Table 3:** Fold change of proteasome subunits upon expression of αSyn or S129A relative to the empty vector (EV) control.

Proteasome Subunit	Description	αSyn/EV (log2)	S129A/EV (log2)
**20S Core:**			
Pre9	Proteasome subunit alpha type-3	−1.48	−0.50
Pre7	Proteasome subunit beta type-6	−1.26	−0.44
Pre8	Proteasome subunit alpha type-2	−1.26	−0.49
Pre10	Proteasome subunit alpha type-7	−1.12	−0.27
Pre3	Proteasome subunit beta type-1	−0.93	−0.46
Pre5	Proteasome subunit alpha type-6	−0.84	−0.25
**19S Regulatory particle—LID:**			
Rpn12	26S proteasome regulatory subunit Rpn12	−1.11	−0.34
Rpn8	26S proteasome regulatory subunit Rpn8	−0.77	−0.30
**19S Regulatory particle—BASE:**			
Rpn13	26S proteasome regulatory subunit Rpn13	−0.92	−0.29
**19S Regulatory particle—ubiquitin receptor:**			
Rpn10	26S proteasome regulatory subunit Rpn10	−0.62	−0.24

## Data Availability

All data generated and analyzed during this study are included in this published article and its Supplementary Information Files.

## Supplementary Materials

The following are available online at https://www.mdpi.com/article/10.3390/cells10092229/s1, Figure S1: Growth effect on yeast cells upon interaction between αSyn and the Tet alleles of the essential genes for proteasome core subunits. Figure S2: Schematic representation of the Bio-ID workflow. Table S1: List of identified proteins with LC-MS analysis. Table S2: List of proteins with significantly different protein abundance upon expression of αSyn in comparison to the empty vector control. Table S3: List of proteins with significantly different protein abundance upon expression of S129A in comparison to the empty vector control. Table S4: Functional enrichment analysis of proteins with changed abundance (αSyn/EV). Table S5: Functional enrichment analysis of proteins with changed abundance (S129A/EV). Table S6: List of proteins significantly enriched in BioID upon expression of αSyn-BirA* or S129A-BirA* in comparison to the BirA* control.
