# Supplementary material for: α-Synuclein Decreases the Abundance of Proteasome Subunits and Alters Ubiquitin Conjugates in Yeast
Source: Cells. 2021 Aug 28;10(9):2229. doi: 10.3390/cells10092229 (PMC8468666; doi:10.3390/cells10092229)
Supplement: Supplementary file 1 [file cells-10-02229-s001.zip › cells-1321177-supplementary/Supplementary Figures S1-S2.pdf]

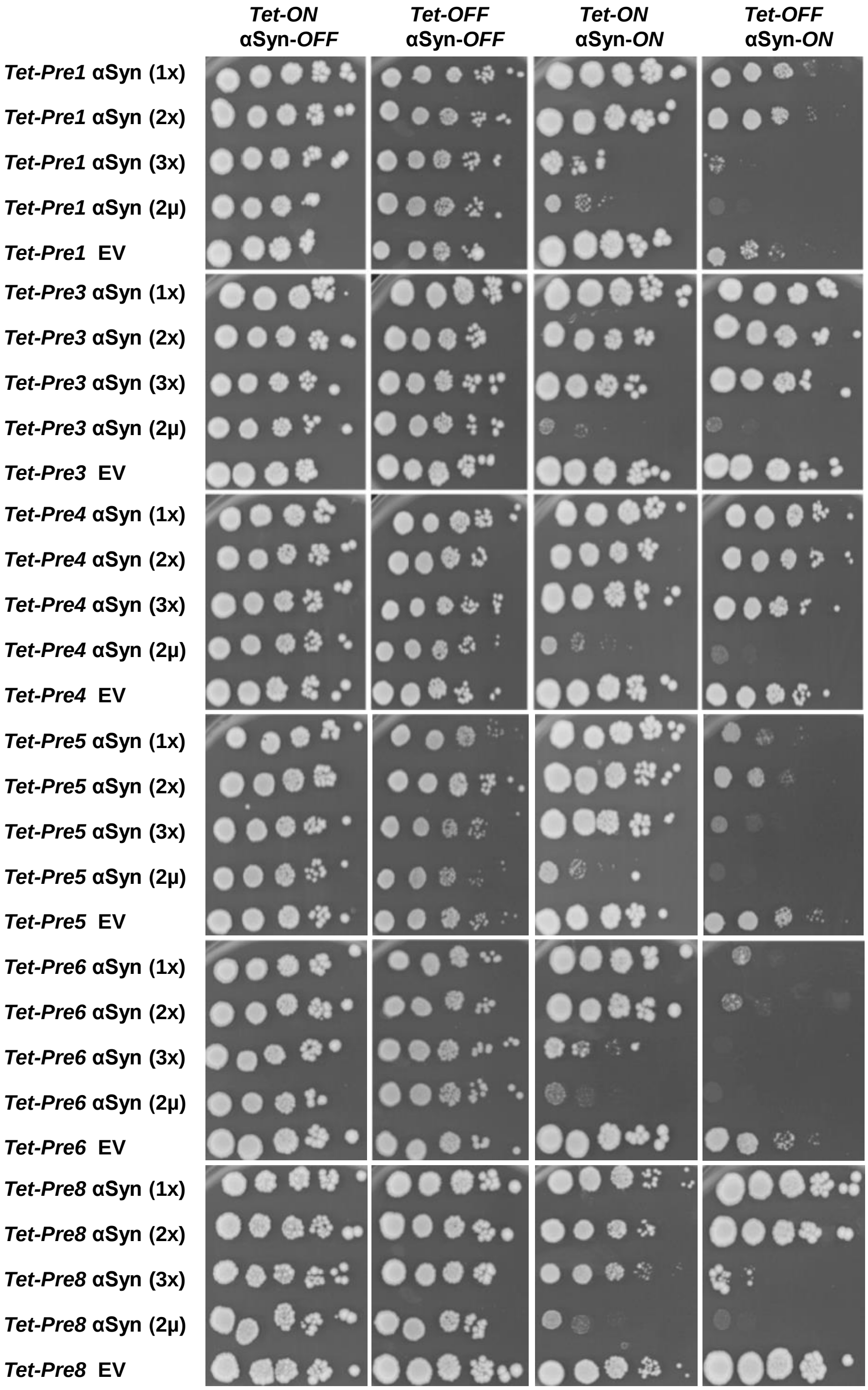

Figure S1. Growth effect on yeast cells upon interaction between αSyn and *Tet*-alleles of essential genes for proteasome core subunits. Growth assays of yeast cells expressing *GAL1*-driven αSyn-GFP from one (1x), two (2x) or three (3x) gene copies or overexpressed from 2µ plasmid with empty vector (EV) as a control. Cells were spotted in 10-fold dilutions on selective plates containing glucose (αSyn-*OFF*) or galactose (αSyn-*ON*), in presence (*Tet-OFF*) or absence (*Tet-ON*) of 10 µg/ml doxycycline that represses the *Tet*-promoter.

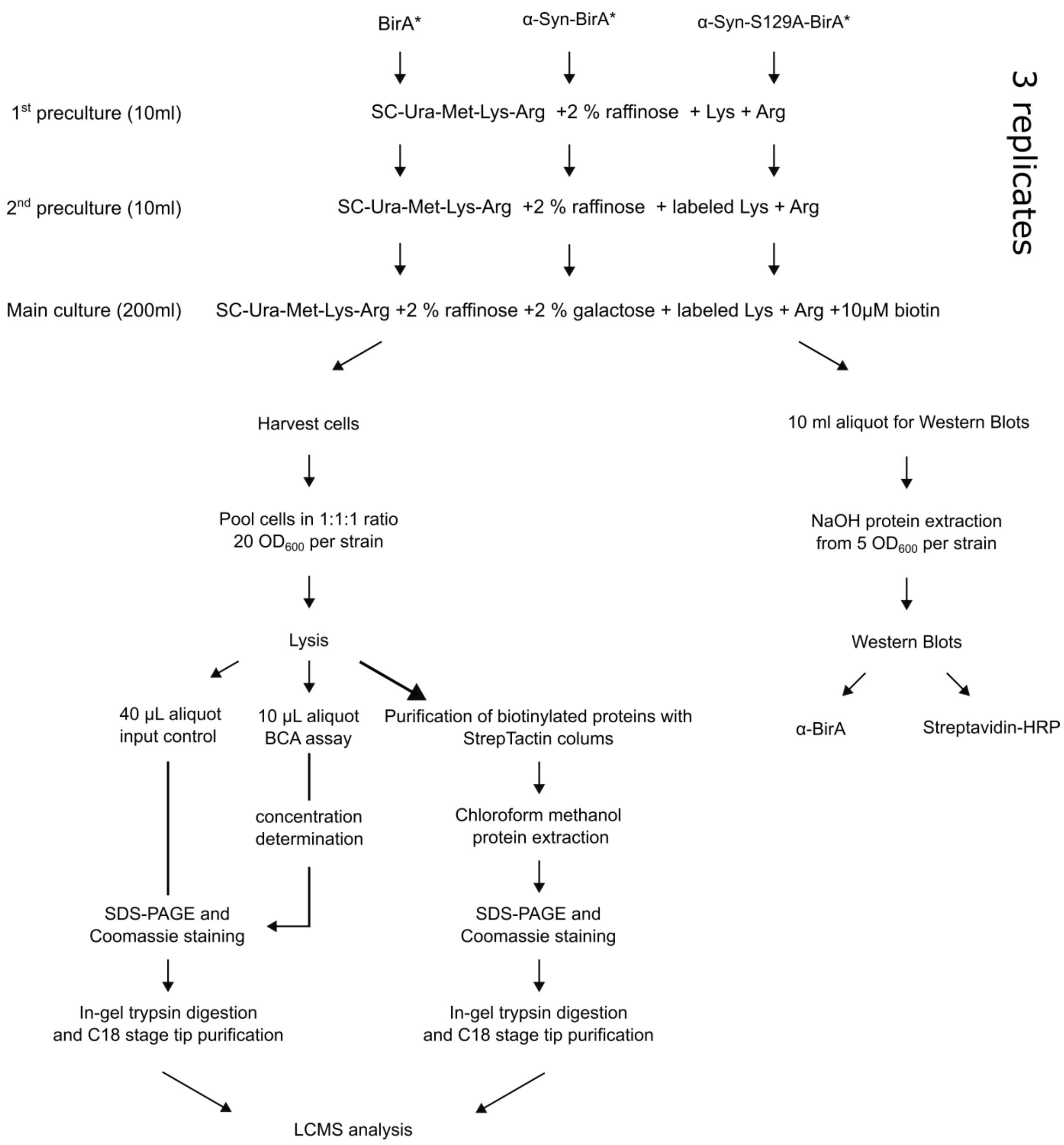

Figure S2. Schematic representation of Bio-ID workflow. For details, see Materials and Methods.
