# Supplementary material for: α-Synuclein Decreases the Abundance of Proteasome Subunits and Alters Ubiquitin Conjugates in Yeast
Source: Cells. 2021 Aug 28;10(9):2229. doi: 10.3390/cells10092229 (PMC8468666; doi:10.3390/cells10092229)
Supplement: Supplementary file 1 [file cells-10-02229-s001.zip › cells-1321177-supplementary/Supplementary Tables S2-S6.pdf]

Table S2. List of proteins with significantly different protein abundance upon expression of aSyn in comparison to empty vector control (log2 aSyn/EV <=-0.7 or >=0.7; p<0.05; n=3).

Gene	Name	log2 aSyn/EV	stddev aSyn/EV
**AAP1**	Alanine/arginine aminopeptidase	-0.85	0.13
**ABP1**	Actin-binding protein	-0.81	0.21
**ACC1**	Acetyl-CoA carboxylase;Biotin carboxylase	0.76	0.09
**ACH1**	Acetyl-CoA hydrolase	-0.88	0.32
**ADH2**	Alcohol dehydrogenase 2	-2.10	0.83
**ADH3**	Alcohol dehydrogenase 3, mitochondrial	-0.80	0.18
**ADK1**	Adenylate kinase	-1.17	0.34
**AIM2**	Protein AIM2	-1.27	0.21
**ALD5**	Aldehyde dehydrogenase 5, mitochondrial	-0.91	0.29
**APE3**	Aminopeptidase Y	-2.15	0.15
**APT1**	Adenine phosphoribosyltransferase 1	-1.44	0.30
**ARC1**	GU4 nucleic-binding protein 1	-1.04	0.31
**ARG7**	Arginine biosynthesis bifunctional protein ArgJ, mitochondrial;	-1.77	0.65
**ARO4**	Phospho-2-dehydro-3-deoxyheptonate aldolase, tyrosine-inhibited	-1.16	0.26
**ASP1**	L-asparaginase 1	-1.00	0.06
**ATP17**	ATP synthase subunit f, mitochondrial	-0.94	0.09
**ATP2**	ATP synthase subunit beta, mitochondrial	-1.00	0.22
**ATP4**	ATP synthase subunit 4, mitochondrial	-0.71	0.14
**ATP5**	ATP synthase subunit 5, mitochondrial	-1.78	0.19
**ATP7**	ATP synthase subunit d, mitochondrial	-0.99	0.12
**BFR1**	Nuclear segregation protein BFR1	-1.82	0.53
**BFR2**	Protein BFR2	-0.74	0.28
**BGL2**	Glucan 1,3-beta-glucosidase	0.90	0.25
**BMH2**	Protein BMH2	-0.73	0.27
**CAJ1**	Protein CAJ1	-1.27	0.29
**CAT2**	Carnitine O-acetyltransferase, mitochondrial	-0.91	0.09
**CCP1**	Cytochrome c peroxidase, mitochondrial	-1.19	0.11
**CDC33**	Eukaryotic translation initiation factor 4E	-0.78	0.16
**CHO2**	Phosphatidylethanolamine N-methyltransferase	0.82	0.20
**CIT2**	Citrate synthase, peroxisomal	-1.23	0.41
**COF1**	Cofilin	-1.10	0.11
**COX4**	Cytochrome c oxidase subunit 4, mitochondrial	-2.69	0.62
**COX5A**	Cytochrome c oxidase polypeptide 5A, mitochondrial	-0.74	0.21
**CPR1**	Peptidyl-prolyl cis-trans isomerase	-1.83	0.37
**CPR3**	Peptidyl-prolyl cis-trans isomerase C, mitochondrial	-2.01	0.36
**CPR5**	Peptidyl-prolyl cis-trans isomerase D	-1.70	0.24
**CRC1**	Mitochondrial carnitine carrier	-1.64	0.40
**CTT1**	Catalase T	-0.98	0.07

**CYB2**	Cytochrome b2, mitochondrial	-1.18	0.35
**CYC1**	Cytochrome c iso-1	-2.71	0.62
**DEF1**	RNA polymerase II degradation factor 1	-1.28	0.42
**DFM1**	DER1-like family member protein 1	0.85	0.19
**EFB1**	Elongation factor 1-beta	-1.90	0.29
**EGD1**	Nascent polypeptide-associated complex subunit beta-1	-2.22	0.29
**EGD2**	Nascent polypeptide-associated complex subunit alpha	-2.42	0.37
**EIS1**	Eisosome protein 1	-1.04	0.06
**ENT2**	Epsin-2	-0.86	0.21
**ERJ5**	ER-localized J domain-containing protein 5	0.79	0.14
**FAS1**	Fatty acid synthase subunit beta;3-hydroxyacyl-[acyl-carrier-protein] dehydratase	0.74	0.09
**FAS2**	Fatty acid synthase subunit alpha	0.77	0.12
**FES1**	Hsp70 nucleotide exchange factor FES1	-0.80	0.14
**FMP52**	Protein FMP52, mitochondrial	-1.09	0.28
**FPR1**	FK506-binding protein 1	-2.13	0.35
**FUN12**	Eukaryotic translation initiation factor 5B	0.70	0.06
**FUR1**	Uracil phosphoribosyltransferase	-0.75	0.05
**GAL2**	Galactose transporter	0.79	0.06
**GAL7**	Galactose-1-phosphate uridylyltransferase	1.08	0.08
**GCV1**	Aminomethyltransferase, mitochondrial	-1.69	0.13
**GCV2**	Glycine dehydrogenase [decarboxylating], mitochondrial	-1.09	0.10
**GDH2**	NAD-specific glutamate dehydrogenase	-0.72	0.27
**GLO1**	Lactoylglutathione lyase	-0.93	0.19
**GLO2**	Hydroxyacylglutathione hydrolase, cytoplasmic isozyme	-0.87	0.26
**GTT1**	Glutathione S-transferase 1	-1.82	0.24
**GUK1**	Guanylate kinase	-1.63	0.30
**HAM1**	Inosine triphosphate pyrophosphatase	-0.83	0.14
**HCR1**	Eukaryotic translation initiation factor 3 subunit J	-1.35	0.36
**HEM15**	Ferrochelatase, mitochondrial	-0.87	0.15
**HEM3**	Porphobilinogen deaminase	-0.84	0.24
**HIS4**	Histidine biosynthesis trifunctional protein;Phosphoribosyl-AMP cyclohydrolase	-1.58	0.59
**HIS5**	Histidinol-phosphate aminotransferase	-0.84	0.12
**HIS7**	Imidazole glycerol phosphate synthase hisHF;Glutamine amidotransferase;Cyclase	-0.80	0.24
**HMF1**	Protein HMF1	-1.09	0.05
**HMO1**	High mobility group protein 1	-1.19	0.32
**HPA2**	Histone acetyltransferase HPA2	-1.95	0.12
**HRB1**	Protein HRB1	-0.77	0.06
**HRP1**	Nuclear polyadenylated RNA-binding protein 4	-1.07	0.35
**HSP10**	10 kDa heat shock protein, mitochondrial	-2.32	0.39
**HSP12**	12 kDa heat shock protein	-2.69	0.72
**HSP150**	Cell wall mannoprotein HSP150	-2.17	0.47
**HSP31**	Probable chaperone protein HSP31	-2.33	0.30

**HSP42**	Heat shock protein 42	-1.11	0.02
**HTA2;HTA1**	Histone H2A.2;Histone H2A.1	-0.73	0.03
**HTB2;HTB1**	Histone H2B.2;Histone H2B.1	-1.05	0.25
**HXK1**	Hexokinase-1	-1.23	0.43
**HYP2**	Eukaryotic translation initiation factor 5A-1	-1.69	0.19
**HYR1**	Peroxiredoxin HYR1	-1.18	0.10
**ICL1**	Isocitrate lyase	-1.93	0.35
**IDP2**	Isocitrate dehydrogenase [NADP] cytoplasmic	-1.36	0.32
**ILV3**	Dihydroxy-acid dehydratase, mitochondrial	-0.82	0.15
**ILV5**	Ketol-acid reductoisomerase, mitochondrial	-1.08	0.28
**ILV6**	Acetolactate synthase small subunit, mitochondrial	-0.96	0.27
**INP53**	Polyphosphatidylinositol phosphatase INP53;SAC1-like phosphoinositide phosphatase	0.71	0.11
**IPP1**	Inorganic pyrophosphatase	-0.87	0.21
**KES1**	Protein KES1	-0.81	0.32
**KRE6**	Beta-glucan synthesis-associated protein KRE6	0.89	0.22
**KTR1**	Alpha-1,2 mannosyltransferase KTR1	1.20	0.18
**LHP1**	La protein homolog	-0.93	0.19
**LSP1**	Sphingolipid long chain base-responsive protein LSP1	-1.94	0.25
**MAM33**	Mitochondrial acidic protein MAM33	-2.13	0.40
**MAP1**	Methionine aminopeptidase 1	-1.16	0.25
**MBF1**	Multiprotein-bridging factor 1	-2.42	0.27
**MDH1**	Malate dehydrogenase, mitochondrial	-1.68	0.30
**MDY2**	Ubiquitin-like protein MDY2	-1.37	0.18
**MET14**	Adenylyl-sulfate kinase	-1.09	0.22
**MET5**	Sulfite reductase [NADPH] subunit beta	0.77	0.24
**MET6**	5-methyltetrahydropteroyltriglutamate--homocysteine methyltransferase	-0.84	0.15
**MGE1**	GrpE protein homolog, mitochondrial	-2.40	0.46
**MLS1**	Malate synthase 1, glyoxysomal	-1.79	0.44
**MMF1**	Protein MMF1, mitochondrial	-2.06	0.15
**MPM1**	Mitochondrial peculiar membrane protein 1	-1.39	0.29
**MRP8**	Uncharacterized protein MRP8	-1.44	0.35
**MRPL1**	54S ribosomal protein L1, mitochondrial	-1.40	0.23
**MRPL13**	54S ribosomal protein L13, mitochondrial	-1.11	0.24
**MRPL15**	54S ribosomal protein L15, mitochondrial	-0.85	0.15
**MRPL40**	54S ribosomal protein L40, mitochondrial	-0.87	0.29
**MRPS28**	37S ribosomal protein S28, mitochondrial	-0.75	0.15
**MSC1**	Meiotic sister chromatid recombination protein 1	-1.38	0.13
**MUP1**	High-affinity methionine permease	2.52	0.48
**NAD(P)H-hydrate**	NAD(P)H-hydrate epimerase	-0.81	0.11
**NFU1**	NifU-like protein, mitochondrial	-1.72	0.42
**NHP2**	H/ACA ribonucleoprotein complex subunit 2	-1.77	0.41
**NOP4**	Nucleolar protein 4	0.94	0.30
**NOP6**	Nucleolar protein 6	-1.12	0.06

**NPL3**	Nucleolar protein 3	-1.04	0.27
**NTF2**	Nuclear transport factor 2	-1.74	0.13
**OM45**	Mitochondrial outer membrane protein OM45	-1.36	0.20
**OST2**	Dolichyl-diphosphooligosaccharide--protein glycosyltransferase subunit OST2	0.95	0.09
**PAA1**	Polyamine N-acetyltransferase 1	0.88	0.35
**PCK1**	Phosphoenolpyruvate carboxykinase [ATP]	-1.11	0.43
**PDX3**	Pyridoxamine 5-phosphate oxidase	-1.51	0.43
**PEP12**	Syntaxin PEP12	-2.60	0.53
**PEP4**	Saccharopepsin	1.10	0.33
**PIL1**	Sphingolipid long chain base-responsive protein PIL1	-1.52	0.40
**PNC1**	Nicotinamidase	-1.26	0.14
**RPL37A**	60S ribosomal protein L37-A	-1.46	0.39
**PNP1**	Purine nucleoside phosphorylase	-0.99	0.34
**POT1**	3-ketoacyl-CoA thiolase, peroxisomal	-1.09	0.37
**PRE10**	Probable proteasome subunit alpha type-7	-1.11	0.30
**PRE3**	Proteasome subunit beta type-1	-0.93	0.24
**PRE5**	Proteasome subunit alpha type-6	-0.84	0.26
**PRE7**	Proteasome subunit beta type-6	-1.26	0.17
**PRE8**	Proteasome subunit alpha type-2	-1.26	0.46
**PRE9**	Proteasome subunit alpha type-3	-1.48	0.37
**PRX1**	Mitochondrial peroxiredoxin PRX1	-1.52	0.21
**QCR7**	Cytochrome b-c1 complex subunit 7	-2.02	0.35
**RAD23**	UV excision repair protein RAD23	-1.41	0.54
**RFA2**	Replication factor A protein 2	-1.70	0.51
**RFA3**	Replication factor A protein 3	-0.94	0.13
**RFS1**	Protein RFS1	-1.04	0.12
**RIB4**	6,7-dimethyl-8-ribityllumazine synthase	0.78	0.28
**RIM1**	Single-stranded DNA-binding protein RIM1, mitochondrial	-3.00	0.37
**RKI1**	Ribose-5-phosphate isomerase	-0.78	0.15
**RPB8**	DNA-directed RNA polymerases I, II, and III subunit RPABC3	-1.65	0.21
**RPE1**	Ribulose-phosphate 3-epimerase	-1.65	0.44
**RPL22A**	60S ribosomal protein L22-A	-1.33	0.26
**RPL22B**	60S ribosomal protein L22-B	-1.17	0.15
**RPL26B;RPL26A**	60S ribosomal protein L26-B;60S ribosomal protein L26-A	-0.92	0.13
**RPL35B;RPL35A**	60S ribosomal protein L35-B;60S ribosomal protein L35-A	-0.98	0.10
**RPN10**	26S proteasome regulatory subunit RPN10	-1.01	0.36
**RPN12**	26S proteasome regulatory subunit RPN12	-0.62	0.27
**RPN13**	26S proteasome regulatory subunit RPN13	-1.11	0.15
**RPN8**	26S proteasome regulatory subunit RPN8	-0.92	0.34
**RPS0B**	40S ribosomal protein S0-B	-0.77	0.37
**RPS10A;RPS10B**	40S ribosomal protein S10-A;40S ribosomal protein S10-B	-0.70	0.22
**RPS19B;RPS19A**	40S ribosomal protein S19-B;40S ribosomal protein S19-A	-1.62	0.44

**RPS22B;RPS22A**	40S ribosomal protein S22-B;40S ribosomal protein S22-A	-0.85	0.16
**RPS25A;RPS25B**	40S ribosomal protein S25-A;40S ribosomal protein S25-B	-0.92	0.18
**RPS26B;RPS26A**	40S ribosomal protein S26-B;40S ribosomal protein S26-A	-0.79	0.15
**RRS1**	Regulator of ribosome biosynthesis	-1.43	0.05
**RSM24**	37S ribosomal protein S24, mitochondrial	-1.61	0.26
**RSM25**	37S ribosomal protein S25, mitochondrial	-0.83	0.27
**RSR1**	Ras-related protein RSR1	-1.18	0.36
**SBA1**	Co-chaperone protein SBA1	0.78	0.13
**SBH1**	Protein transport protein SBH1	-2.01	0.40
**SCD6**	Protein SCD6	0.84	0.31
**SDH2**	Succinate dehydrogenase [ubiquinone] iron-sulfur subunit, mitochondrial	-1.14	0.37
**SEC28**	Coatomer subunit epsilon	-1.23	0.30
**SFC1**	Succinate/fumarate mitochondrial transporter	-1.23	0.38
**SGT2**	Small glutamine-rich tetratricopeptide repeat-containing protein 2	-1.85	0.20
**SHM2**	Serine hydroxymethyltransferase, cytosolic	-1.22	0.32
**SHP1**	UBX domain-containing protein 1	-0.80	0.17
**SNC2**	Synaptobrevin homolog 2	-1.12	0.34
**SNF7**	Vacuolar-sorting protein SNF7	0.79	0.27
**SNU13**	13 kDa ribonucleoprotein-associated protein	-1.20	0.34
**SNZ1**	Pyridoxine biosynthesis protein SNZ1	-1.92	0.23
**SOD1**	Superoxide dismutase [Cu-Zn]	-3.14	0.69
**SOD2**	Superoxide dismutase [Mn], mitochondrial	-3.56	0.61
**SOL4**	6-phosphogluconolactonase 4	-2.16	0.30
**SPT15**	TATA-box-binding protein	-0.85	0.17
**SSE2**	Heat shock protein homolog SSE2	0.79	0.24
**STM1**	Suppressor protein STM1	-0.74	0.26
**SUA7**	Transcription initiation factor IIB	-1.26	0.28
**TDH1**	Glyceraldehyde-3-phosphate dehydrogenase 1	0.74	0.28
**TFS1**	Carboxypeptidase Y inhibitor	0.79	0.15
**THO1**	Protein THO1	-1.68	0.23
**TIF11**	Eukaryotic translation initiation factor 1A	-2.06	0.41
**TIM11**	ATP synthase subunit e, mitochondrial	-1.45	0.24
**TMA19**	Translationally-controlled tumor protein homolog	-0.78	0.21
**TPM1**	Tropomyosin-1	-1.67	0.22
**TRX1**	Thioredoxin-1	-2.48	0.70
**TRX2**	Thioredoxin-2	-2.10	0.42
**TSA1**	Peroxiredoxin TSA1	-1.97	0.60
**TY2B-C**	Transposon Ty2-C Gag-Pol polyprotein;Capsid protein;	-1.19	0.32
**UBC1**	Ubiquitin-conjugating enzyme E2 1	0.76	0.23
**UBC13**	Ubiquitin-conjugating enzyme E2 13	-0.78	0.10
**URA1**	Dihydroorotate dehydrogenase (fumarate)	-0.79	0.20
**URA2**	Protein URA2;Glutamine-dependent carbamoyl-phosphate synthase	1.68	0.18

**URA3**	Orotidine 5-phosphate decarboxylase	1.90	0.18
**URA8**	CTP synthase 2	-3.24	0.34
**UTR4**	Enolase-phosphatase E1	1.29	0.33
**VMA4**	V-type proton ATPase subunit E	-1.19	0.27
**VPS21**	Vacuolar protein sorting-associated protein 21	-1.30	0.31
**YAT2**	Carnitine O-acetyltransferase YAT2	0.91	0.31
**YGL101W**	HD domain-containing protein YGL101W	-1.91	0.46
**YGP1**	Protein YGP1	-0.84	0.12
**YHB1**	Flavohemoprotein	-1.23	0.32
**YHI9**	Uncharacterized isomerase YHI9	-0.93	0.24
**YIP3**	Prenylated Rab acceptor 1	-1.77	0.66
**YJL055W**	LOG family protein YJL055W	1.13	0.15
**YKL033W-A**	Putative uncharacterized hydrolase YKL033W-A	-1.10	0.29
**YLR179C**	Uncharacterized protein YLR179C	-1.33	0.48
**YML079W**	Uncharacterized protein YML079W	-2.16	0.56
**YMR178W**	Uncharacterized protein YMR178W	-1.24	0.16
**YMR226C**	Uncharacterized oxidoreductase YMR226C	-0.97	0.11
**YNL208W**	Uncharacterized protein YNL208W	-1.86	0.50
**YOS1**	Protein transport protein YOS1	-2.24	0.67
**ORM2**	Protein ORM2	0.82	0.15
**YPR114W**	Uncharacterized TLC domain-containing protein YPR114W	0.84	0.08
**YPR172W**	Pyridoxamine 5-phosphate oxidase homolog	-1.03	0.27
**YPS1**	Aspartic proteinase 3;Aspartic proteinase 3 subunit alpha;Aspartic proteinase 3 subunit beta	0.72	0.18
**YPT1**	GTP-binding protein YPT1	0.72	0.19
**YRB1**	Ran-specific GTPase-activating protein 1	-1.46	0.36
**YTM1**	Ribosome biogenesis protein YTM1	-0.72	0.09
**ZEO1**	Protein ZEO1	-2.56	0.45
**ZTA1**	Probable quinone oxidoreductase	-1.73	0.34

Table S3. List of proteins with significantly different protein abundance upon expression of S129A in comparison to empty vector control (log2 S129A/EV <= -0.7 or >=0.7; p<0.05; n=3).

Gene	Name	log2 S129A/EV	stddev S129A/EV
**ALD3**	Aldehyde dehydrogenase [NAD(P)+] 2	0.73	0.29
**APE3**	Aminopeptidase Y	-0.89	0.28
**ATP5**	ATP synthase subunit 5, mitochondrial	-0.75	0.19
**BGL2**	Glucan 1,3-beta-glucosidase	0.82	0.29
**CYB2**	Cytochrome b2, mitochondrial	-0.70	0.27
**EGD2**	Nascent polypeptide-associated complex subunit alpha	-0.95	0.22
**HSP10**	10 kDa heat shock protein, mitochondrial	-1.33	0.46
**HSP150**	Cell wall mannoprotein HSP150	-1.06	0.25
**HXT6;HXT7**	High-affinity hexose transporter HXT6	-1.37	0.20
**HYP2**	Eukaryotic translation initiation factor 5A-1	-0.77	0.28
**KTR1**	Alpha-1,2 mannosyltransferase KTR1	0.90	0.17
**MAM33**	Mitochondrial acidic protein MAM33	-1.05	0.17
**MMF1**	Protein MMF1, mitochondrial	-1.00	0.39
**MRPL1**	54S ribosomal protein L1, mitochondrial	-0.72	0.08
**MUP1**	High-affinity methionine permease	0.71	0.15
**NFU1**	NifU-like protein, mitochondrial	-1.01	0.33
**NHP2**	H/ACA ribonucleoprotein complex subunit 2	-0.75	0.29
**RIM1**	Single-stranded DNA-binding protein RIM1, mitochondrial	-0.90	0.28
**RPL37A**	60S ribosomal protein L37-A	-0.74	0.16
**RRS1**	Regulator of ribosome biosynthesis	-0.85	0.29
**TMA19**	Translationally-controlled tumor protein homolog	-0.78	0.19
**TPM1**	Tropomyosin-1	-0.82	0.18
**TRX1**	Thioredoxin-1	-1.08	0.40
**URA3**	Orotidine 5-phosphate decarboxylase	-1.29	0.14
**YIP3**	Prenylated Rab acceptor 1	0.70	0.16
**YMR226C**	Uncharacterized oxidoreductase YMR226C	-0.74	0.27

Table S4. Functional enrichment analysis of proteins with changed abundance (αSyn/EV). Protein enrichment analysis was carried out using FunSpec webserver (1). Proteins were classified in functional categories or subcellular localization using MIPS (Munich Information Center for Protein Sequences) functional classification. Proteins with increased or decreased abundance in comparison to the control are analysed separately. "k": number of genes from the input cluster in given category; "f": total number of genes in given category.

**MIPS Functional Classification**				
**aSyn/EV decreased abundance**				
**Category**	**p-value**	**In Category from Cluster**	**k**	**f**
protein processing (proteolytic) [14.07.11]	3.00E-08	PRE7 APE3 RPN12 PRE9 AAP1 RPN10 PRE3 MAP1 RPN13 PRE8 PRE5 PRE10 PEP4	13	63
electron transport and membrane-associated energy conservation [02.11]	1.03E-08	ATP5 TIM11 ATP17 QCR7 ALD5 COX4 CYC1 ATP2 ATP7 SDH2 CYB2 COX5A ATP4	13	58
electron transport [20.01.15]	1.29E-07	ATP5 TIM11 ATP17 COX4 TRX2 CYC1 ATP2 ATP7 CCP1 SDH2 TRX1 COX5A VMA4 ATP4	14	83
oxidative stress response [32.01.01]	5.04E-07	PRX1 HSP12 CTT1 TRX2 SOD2 HYR1 GTT1 SOD1 CCP1 TRX1 TSA1	11	55
energy generation (e.g. ATP synthase) [02.45.15]	2.51E-05	ATP5 TIM11 ATP17 ATP2 ATP7 ATP4	6	21
proteasomal degradation (ubiquitin/proteasomal pathway) [14.13.01.01]	2.58E-05	PRE7 SHP1 YRB1 UBC13 UBC1 RPN12 PRE9 RPN10 PRE3 DEF1 PRE8 PRE5 RPN8 PRE10	14	128
ribosomal proteins [12.01.01]	0.000132	RPL35B MRPL1 RSM24 MRPS28 RPS26B RPL22B RPS25A RPL26B RSM25 MRP8 MRPL13 RPS0B RPL22A RPL37A MRPL15 RPS22B RPS19B RPS10A MRPL40	19	246
metabolism of vitamins, cofactors, and prosthetic groups [01.07]	0.0002526	ACH1 GCV1 PNC1 GTT1 SHM2 GLO1 GCV2	7	43
biosynthesis of isoleucine [01.01.11.02.01]	0.000254	MMF1 ILV3 ILV5	3	5
oxygen and radical detoxification [32.07.07]	0.0003214	PRX1 TRX2 TRX1 TSA1	4	12
respiration [02.13]	0.0003384	ATP5 TIM11 ATP17 CYC1 ATP2 ATP7 CYB2 ATP4	8	59
protein folding and stabilization [14.01]	0.0004334	SSE2 CPR1 HSP42 CPR5 CAJ1 SBA1 CPR3 FPR1 HSP10 MGE1	10	93
degradation of glycine [01.01.09.01.02]	0.0004969	GCV1 SHM2 GCV2	3	6
biosynthesis of valine [01.01.11.03.01]	0.0004969	ILV6 ILV3 ILV5	3	6
superoxide metabolism [32.07.07.07]	0.0008948	SOD2 SOD1	2	2
mitochondrial transport [20.09.04]	0.001052	ATP5 TIM11 YAT2 SFC1 ATP2 ATP7 CAT2 CRC1 MGE1 ATP4	10	104
mitochondrion [42.16]	0.0017	MRPL1 RSM24 MRPS28 ALD5 MMF1 RSM25 MDH1 MRPL13 CCP1 MRPL15 CYB2 HSP10 MRPL40	13	170

glyoxylate cycle [02.04]	0.001952	CIT2 ICL1 MLS1	3	9
tricarboxylic-acid pathway (citrate cycle, Krebs cycle, TCA cycle) [02.10]	0.002075	CIT2 GDH2 MDH1 SDH2 IDP2	5	31
metabolism of nonprotein amino acids [01.20.17.01]	0.002727	YAT2 CAT2 ARG7	3	10
C-compound and carbohydrate metabolism [01.05]	0.006917	ACH1 ILV6 CIT2 GLO2 YAT2 ICL1 HSP12 HXK1 PCK1 IDP2 GLO1 CYB2 ADH3 MLS1	14	223
aerobic respiration [02.13.03]	0.008001	MRPL1 RSM24 QCR7 COX4 MAM33 SDH2 COX5A	7	77
protease inhibitor [18.02.01.02.03]	0.008428	RAD23 TFS1	2	5
C-1 compound catabolism [01.05.05.07]	0.008428	GCV1 GCV2	2	5
biosynthesis of histidine [01.01.09.07.01]	0.009258	HIS7 HIS4 HIS5	3	15
**MIPS Functional Classification**				
**aSyn/EV increased abundance**				
**Category**	**p-value**	**In Category from Cluster**	**k**	**f**
fatty acid metabolism [01.06.05]	0.0002559	FAS1 ACC1 FAS2	3	24
pyrimidine nucleotide/nucleoside/nucleobase metabolism [01.03.04]	0.000554	URA2 URA8 URA1	3	31
endocytosis [20.09.18.09.01]	0.00362	VPS21 INP53 SNC2	3	59
cell wall [42.01]	0.004913	BGL2 MET5 KTR1 INP53 KRE6	5	213
ER to Golgi transport [20.09.07.03]	0.005859	YOS1 YPT1 YIP3	3	70
sugar, glucoside, polyol and carboxylate catabolism [01.05.02.07]	0.008776	GAL7 TDH1 KRE6	3	81

**MIPS Subcellular Localization**				
**aSyn/EV decreased abundance**				
**Category**	**p-value**	**In Category from Cluster**		

cytoplasm [725]	2.68E-11	EFB1 AIM2 ACH1 PRE7 SHP1 PRX1 IPP1 PDX3 ZTA1 FES1 SSE2 HIS7 ARO4 HIS4 CIT2 ABP1 LHP1 RPL35B HEM3 NHP2 NOP6 GDH2 YRB1 PAA1 UBC13 BMH2 CPR1 HSP42 RSM24 UBC1 ADK1 GLO2 CPR5 ASP1 MRPS28 GUK1 HSP31 HYP2 RAD23 UTR4 FMP52 NTF2 YAT2 HMF1 ICL1 MET6 RPS26B HSP12 RPL22B HXK1 PNC1 YGL101W ARC1 RPS25A RPL26B PIL1 CTT1 PRE9 TRX2 YHB1 SOL4 YHI9 AAP1 FUR1 EGD2 RPN10 HIS5 POT1 HYR1 YJL055W MPM1 RPE1 HSP150 CYC1 HAM1 SFC1 SOD1 MET14 DEF1 TMA19 SBA1 MRP8 PCK1 TRX1 RPS0B SHM2 RPL22A STM1 IDP2 TFS1 YLR179C RPL37A HCR1 MAP1 RPS22B RPN13 GLO1 APT1 TSA1 YML079W MSC1 SNZ1 YMR178W YMR226C TIF11 ADH2 PRE5 TPM1 MLS1 FPR1 YGP1 YNL208W RPS19B RFA2 ZEO1 MDY2 HRP1 CDC33 SGT2 RKI1 MGE1 RPN8 RPS10A RRS1 MBF1 PRE10 LSP1 EGD1 KES1 SCD6 YPR172W HPA2	**k**	**f**
mitochondria [755]	1.50E-10	ACH1 PRX1 SSE2 ILV6 CIT2 RIM1 GDH2 GCV1 MRPL1 RSM24 ADK1 ATP5 TIM11 MRPS28 ATP17 QCR7 HYP2 RAD23 FMP52 YAT2 ALD5 COX4 PIL1 YHB1 SOD2 MMF1 MAM33 RSM25 OM45 GTT1 YJL055W MPM1 ILV3 CYC1 SFC1 SOD1 ATP2 ATP7 NFU1 MDH1 MRP8 MRPL13 CCP1 SDH2 PNP1 MRPL15 ILV5 CAT2 CYB2 CPR3 MSC1 EIS1 ARG7 ADH3 GCV2 COX5A YNL208W ZEO1 HSP10 CRC1 HEM15 MGE1 MBF1 LSP1 ATP4 PEP4 MRPL40	132	2879
mitochondrial matrix [755.07]	0.0002434	ACH1 PRX1 RIM1 MRPL1 SOD2 MAM33 RSM25 NFU1 ARG7	67	1042
mitochondrial inner membrane [755.05]	0.0005293	ATP5 TIM11 QCR7 COX4 SFC1 ATP2 ATP7 CCP1 SDH2 COX5A CRC1 HEM15 ATP4	9	71

nucleus [750]	0.001509	AIM2 HTB2 HTA2 PRE7 SHP1 PRX1 IPP1 ZTA1 HIS7 ARO4 LHP1 HEM3 NHP2 NOP6 UBC13 BMH2 CPR1 HMO1 UBC1 ADK1 GLO2 NPL3 GUK1 HSP31 SNU13 RAD23 UTR4 NTF2 HMF1 THO1 HSP12 RPN12 YGL101W PRE9 TRX2 SOL4 AAP1 RPN10 PRE3 YJL055W RPE1 RFA3 CYC1 HAM1 SOD1 DEF1 SBA1 MRPL13 TRX1 SHM2 TFS1 YLR179C PNP1 MAP1 RPN13 GLO1 APT1 CYB2 YML079W PRE8 YMR178W YMR226C PRE5 HRB1 TPM1 FPR1 RFA2 HRP1 RKI1 RPB8 RPN8 YTM1 RPS10A RRS1 MBF1 PRE10 EGD1 KES1 YPR172W	13	150
peroxisomal matrix [760.03]	0.002134	CIT2 POT1 CAT2 MLS1	79	1976
**MIPS Subcellular Localization**				
**aSyn/EV increased abundance**				
**Category**	**p-value**	**In Category from Cluster**	**k**	**f**
ER [735]	2.78E-09	DFM1 SBH1 YPT1 ERJ5 CHO2 BGL2 YPS1 ORM2 YIP3 ACC1 PEP12 VPS21 KTR1 OST2 SNC2 YPR114W	16	537
vacuole [770]	0.001013	MUP1 BGL2 PEP12 KTR1 SNC2 KRE6	6	224
golgi membrane [740.01]	0.001496	YOS1 YPT1 YIP3 KTR1	4	95
nuclear envelope [750.01]	0.001711	BGL2 ACC1 PEP12 KTR1 SNC2	5	167
golgi [740]	0.00407	YPT1 YIP3 KTR1 KRE6	4	125
ER membrane [735.01]	0.004812	YOS1 SBH1 ACC1 OST2	4	131

Table S5. Functional enrichment analysis of proteins with changed abundance (S129A/EV). Protein enrichment analysis was carried out using FunSpec webserver. Proteins were classified in functional categories or subcellular localization using MIPS (Munich Information Center for Protein Sequences) functional classification. Proteins with increased or decreased abundance in comparison to the control were analysed separately. ”k”: number of genes from the input cluster in given category; “f”: total number of genes in given category.

**MIPS Functional Classification**				
**S129A/EV decreased abundance**				
**Category**	**p-value**	**In Category from Cluster**	**k**	**f**
mitochondrion [42.16]	0.00148<br>8	MRPL1 MMF1 CYB2 HSP10	4	170
sugar transport [20.01.03.01]	0.00464<br>8	HXT7 HXT6	2	31
**MIPS Functional Classification**				
**S129A/EV increased abundance**				
**Category**	**p-value**	**In Category from Cluster**	**k**	**f**
C-compound and carbohydrate metabolism [01.05]	0.00036<br>15	BGL2 ALD3 KTR1	3	223
aldehyde dehydrogenase [NAD(P)+] activity	0.00302<br>6	ALD3	1	4
cell wall [42.01]	0.00971<br>2	BGL2 KTR1	2	213

**MIPS Subcellular Localization**				
**S129A/EV decreased abundance**				
**Category**	**p-value**	**In Category from Cluster**	**k**	**f**
mitochondrial matrix [755.07]	7.76E-<br>05	RIM1 MRPL1 MAM33 NFU1	4	71
mitochondria [755]	0.00018<br>78	RIM1 MRPL1 ATP5 HXT7 HXT6 HYP2 MMF1 MAM33 NFU1 CYB2 HSP10	11	1042
**MIPS Subcellular Localization**				
**S129A/EV increased abundance**				
**Category**	**p-value**	**In Category from Cluster**	**k**	**f**
vacuole [770]	0.00036<br>63	MUP1 BGL2 KTR1	3	224
golgi membrane [740.01]	0.00199<br>1	YIP3 KTR1	2	95
golgi [740]	0.00342<br>5	YIP3 KTR1	2	125
ER [735]	0.00472<br>3	BGL2 YIP3 KTR1	3	537
nuclear envelope [750.01]	0.00604<br>7	BGL2 KTR1	2	167

Table S6. List of proteins significantly enriched in BioID upon expression of αSyn-BirA* or S129A-BirA* in comparison to BirA* control. Following thresholds were applied: one sample t-test $p<0.05$; log2 SILAC ratio > 0.7. The values are mean and standard deviation (SD) from three independent experiments. Genes were catalogued according to their function and subcellular localization using criteria from the Saccharomyces Genome Database (SGD) and manual annotation. PM - plasma membrane; ER - endoplasmic reticulum.

Gene names	Protein names	αSyn/ BirA mean	αSyn/ BirA SD	S129A/ BirA mean	S129A/ BirA SD	Category/ function	Subcellular localization
**AIM13**	**Altered inheritance of mitochondria protein 13, mitochondrial**	1.50	0.14	1.66	0.30	compartments	mitochondria
**ALR1**	**Magnesium transporter ALR1**	2.02	0.42	1.89	0.67	transmembrane transport	PM, membrane
**APM4**	**AP-2 complex subunit mu**	0.69	0.63	0.90	0.24	transport/ endocytosis	PM, membrane, vesicle
**BOI1**	**Protein BOB1**	0.77	0.37	1.23	0.15	transport/ vesicle organization	PM, Golgi, cytoplasm
**BUD2**	**Inhibitory regulator protein BUD2/CLA2**	1.61	0.22	1.65	0.43	Cell cycle	membrane, cytoplasm
**BUD3**	**Bud site selection protein 3**	0.92	0.33	1.24	0.14	Cell cycle	membrane, cytoplasm
**CDC12**	**Cell division control protein 12**	0.95	0.22	1.07	0.32	Cell cycle	membrane, cytoplasm
**CRP1**	**Cruciform DNA-recognizing protein 1**	1.61	0.03	1.79	0.24	DNA binding	cytoplasm, nucleus
**CSR2**	**Transcription factor CSR2**	1.56	0.29	1.53	0.81	transcription	cytoplasm, nucleus
**EFR3**	**Protein EFR3**	1.28	0.29	1.58	0.54	Kinase localization at plasma membrane	PM
**GIN4**	**Serine/threonine-protein kinase GIN4**	0.77	0.14	0.88	0.40	Signalling	cytoplasm, nucleus
**GPA1**	**Guanine nucleotide-binding protein alpha-1 subunit**	2.20	0.76	2.38	0.22	Signalling	PM
**HXT6; HXT7**	**High-affinity hexose transporter HXT6**	2.35	0.32	2.68	0.21	transmembrane transport	PM, membrane, mitochondria
**IST2**	**Increased sodium tolerance protein 2**	1.83	0.24	2.28	0.54	transmembrane transport	PM, ER
**LRG1**	**Rho-GTPase-activating protein LRG1**	1.42	0.13	1.56	0.39	Signalling	cytopalsm, mitochondria
**MDG1**	**Signal transduction protein MDG1**	1.70	0.30	2.11	0.33	Signalling	PM, cytopalsm, nucleus
**OSH2**	**Oxysterol-binding protein homolog 2**	1.69	0.15	1.86	0.38	transport/ endocytosis	PM, ER
**OSH3**	**Oxysterol-binding protein homolog 3**	1.05	0.28	1.43	0.31	transport/ endocytosis	PM, ER
**PAM1**	**Protein PAM1**	1.09	0.43	1.56	0.53	morphogenesis	membrane, cytoplasm
**PMA1; PMA2**	**Plasma membrane ATPase 1;Plasma membrane ATPase 2**	0.82	0.29	1.09	0.43	transmembrane transport	PM, cytopalsm, mitochondria
**PPZ1**	**Serine/threonine-protein phosphatase PP-Z1**	1.06	0.30	1.41	0.51	Signalling	PM, cytopalsm, nucleus
**RAS1**	**Ras-like protein 1**	2.16	0.84	2.25	0.65	Signalling	membrane, mytochondria, nucleus
**RAS2**	**Ras-like protein 2**	2.87	0.47	3.14	0.31	Signalling	ER, membrane,

							mytochondria, nucleus
**RFS1**	**Protein RFS1**	1.62	0.50	1.79	0.28	unknown	membrane, cytoplasm
**RHO2**	**GTP-binding protein RHO2**	2.87	0.31	3.12	0.17	Signalling	membrane, vesicle, Golgi
**ROM2**	**RHO1 GDP-GTP exchange protein 2**	0.97	0.42	1.34	0.41	Signalling	nucleus
**RPT2**	**26S protease regulatory subunit 4 homolog**	0.71	0.10	0.49	0.48	Protein stability 26S Proteasome	cytoplasm, nucleus
**RSR1**	**Ras-related protein RSR1**	3.30	0.44	3.36	0.33	Signalling	ER, membrane
**RTK1**	**Probable serine/threonine-protein kinase RTK1**	1.80	0.29	2.15	0.58	Signalling	cytoplasm, nucleus
**SEC4**	**Ras-related protein SEC4**	0.62	0.13	0.85	0.12	Signalling	ER, Golgi, vesicle, membrane, cytoplasm
**SKG3**	**Protein SKG3**	0.37	0.19	0.85	0.30	Unknown / cell periphery	cell periphery
**SLM1**	**Phosphatidylinositol 4,5-bisphosphate-binding protein SLM1**	1.71	0.28	1.94	0.45	Actin cytoskeleton	membrane, mitochondria
**SLM2**	**Phosphatidylinositol 4,5-bisphosphate-binding protein SLM2**	1.30	0.16	1.68	0.50	Actin cytoskeleton	PM
**SYP1**	**Suppressor of yeast profilin deletion**	1.88	0.19	2.25	0.51	Endocytosis	membrane, cytoplasm, vesicle
**TCB1**	**Tricalbin-1**	1.24	0.43	1.44	0.51	ER membrane organization	ER, membrane
**TCB3**	**Tricalbin-3**	1.65	0.26	1.98	0.40	ER membrane organization	ER, membrane
**YBL086C**	**Uncharacterized protein YBL086C**	1.37	0.46	1.81	0.31	Unknown/cell periphery	cell periphery
**YCK1**	**Casein kinase I homolog 1**	2.90	0.32	3.23	0.20	Signalling	ER, membrane, mitochondria, nucleus
**YCK2**	**Casein kinase I homolog 2**	2.83	0.50	3.28	0.19	Signalling	membrane, mitochondria, nucleus
**YCP4**	**Flavoprotein-like protein YCP4**	1.92	0.25	1.99	0.12	Unknown/mitochondria	membrane, mitochondria
**YGL082W**	**Uncharacterized protein YGL082W**	1.03	0.36	1.19	0.06	Protein stability putative Mindy deubiquitinase	PM, cytoplasm, nucleus
**YKT6**	**Synaptobrevin homolog YKT6**	1.48	0.57	1.76	0.44	Golgi vesicle transport	Golgi, PM, membrane,
**YPL199C**	**Smr domain-containing protein YPL199C**	3.00	0.14	3.10	0.20	mRNA decay	cytoplasm
**YPT10**	**GTP-binding protein YPT10**	1.39	0.63	1.58	0.42	Signalling	membrane, cytoplasm, vesicle

1. Robinson, M. D., Grigull, J., Mohammad, N., and Hughes, T. R. (2002) FunSpec: A web-based cluster interpreter for yeast. *BMC Bioinformatics*. 10.1186/1471-2105-3-35
